# A Fast-Pass, Desorption Electrospray Ionization Mass Spectrometry Strategy for Untargeted Metabolic Phenotyping

**DOI:** 10.1021/jasms.4c00459

**Published:** 2025-01-27

**Authors:** Hawkins S. Shepard, Jody C. May, Baltazar E. Zuniga, Joshua P. Abraham, Brian F. Pfleger, Jamey D. Young, John A. McLean

**Affiliations:** 1 Department of Chemistry, Center for Innovative Technology, Vanderbilt University, Nashville, Tennessee 37235, United States; 2 Department of Chemical and Biomolecular Engineering, Vanderbilt University, Nashville, Tennessee 37235, United States; 3 Department of Chemical and Biological Engineering, University of Wisconsin, Madison, Wisconsin 53706, United States; 4 Department of Molecular Physiology and Biophysics, Vanderbilt University, Nashville, Tennessee 37235, United States

**Keywords:** DESI-MS, mass spectrometry imaging, synthetic biology, molecular engineering, lipidomics, metabolomics

## Abstract

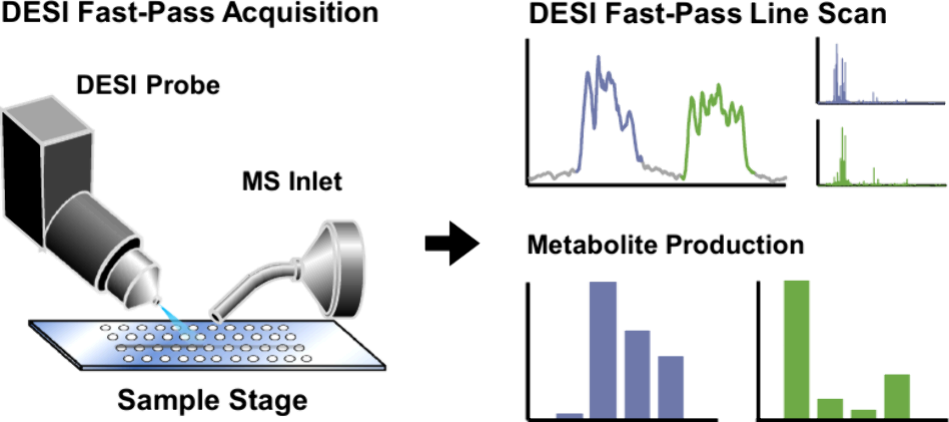

Desorption electrospray ionization mass spectrometry imaging (DESI-MSI) provides direct analytical readouts of small molecules that can be used to characterize the metabolic phenotypes of genetically engineered bacteria. In an effort to accelerate the time frame associated with the screening of mutant libraries, we have developed a high-throughput DESI-MSI analytical workflow implementing a single raster line-scan strategy that facilitates the collection of location-resolved molecular information from engineered strains on a subminute time scale. Evaluation of this “Fast-Pass” DESI-MSI phenotyping workflow on analytical standards demonstrated the capability of acquiring full metabolic profiling information with a throughput of ∼40 s per sample. This Fast-Pass strategy was implemented in the analysis of genetically edited *Escherichia coli* strains that have been engineered to produce various free-fatty acids (FFAs) for applications relevant to biofuels. Due to the untargeted nature of DESI-MSI, the investigation of these strains yielded molecular information for both global metabolites and targeted detection of accumulated bioproducts, allowing simultaneous readouts of strain-specific chemical profiles and comparative measurements of FFA production levels.

## Introduction

Microbially derived natural products have historically held economic and cultural significance due to their importance in numerous industries such as bacterial fermentation (e.g., ethanol, lactate, and amino acids) and fungal antibiotics (e.g., penicillins and cephalosporins). In the second half of the 20th century, discoveries such as recombinant DNA and polymerase chain reaction laid the foundation for genetic engineering by providing researchers with tools to directly modify and program bacterial systems through genomic manipulation.^1^ More recently, innovations including multiplexed automated genome engineering and CRISPR-Cas9 gene editing technology have significantly empowered metabolic engineering by enabling more precise manipulation of microbial systems on a genetic level, targeting enhanced production of high-value commodity chemicals as well as the potential introduction of non-native synthetic pathways.^2−4^ Synthetic biology strategies are now able to produce genetically engineered microorganisms faster than these modified strains can be characterized by traditional bioanalytical workflows.^5^ The rate-limiting step for metabolic engineering is no longer in the reading and writing of DNA but rather in determining which sequences are optimal to write.

Frequently, the objective of genetic engineering is to promote the biosynthesis of high-value chemical products by relatively low-cost microbial cultures such as the production of FFAs in *E. coli* and cyanobacteria. By manipulating fatty acid biosynthesis in *E. coli* through the expression of different thioesterase enzymes of various strengths, new strains can be developed with curated FFA production profiles.^6−9^ These genetically altered microbes have the potential to replace traditional fossil fuel feedstocks and become a renewable platform for the on-site and on-demand synthesis of surfactants, lubricants and biofuel precursors.^10^ However, even carefully chosen gene edits can have unpredictable broad scale effects on the microbial metabolome. As such, untargeted chemical analyses are particularly useful, as they can provide global metabolite profiles including information regarding the levels of pathway intermediates and upstream precursors, potential off-target effects on other pathways, and accumulation of desired products as well as unwanted byproducts.

Mass spectrometry (MS) based workflows are often implemented in phenomic profiling due to the ability to leverage the high sensitivity, specificity, and speed of MS toward the study of small biomolecules via metabolomic and lipidomic analyses.^11^ Established methodologies that incorporate liquid chromatography (LC) and gas chromatography (GC) separations coupled to MS benefit from increased peak capacity, which improves metabolic specificity and coverage; however, the lengthy analysis times associated with the chromatography step result in relatively low sample throughput (∼100 samples/day) that is insufficient to meet the screening requirements of current genetic engineering strategies.^12,13^ Workflows using matrix assisted laser desorption ionization (MALDI) MS have been demonstrated to increase throughput and decrease sample preparation requirements; however, matrix interferences and compatibility issues associated with the requirement for vacuum-based sampling hinder the analysis of more labile and volatile analytes such as those of interest for biofuel applications.^14−17^ Recently, desorption electrospray ionization-MS imaging (DESI-MSI) was demonstrated as a high-throughput screening strategy for its ability to acquire direct metabolic readouts of samples under ambient conditions while operating in a spatially resolved manner.^18^ By incorporating DESI-MSI in untargeted screening workflows, the time frame associated with phenotyping can be decreased by more than an order of magnitude in comparison to chromatography-based (LC–MS or GC–MS) strategies. This increase in throughput can also be translated to the data analysis level (typically a bottleneck in these workflows) by performing unsupervised segmentation for the *in situ* differentiation of strains in a multiplexed manner and decreasing sample handling by growing and imaging bacteria on the same microporous membrane substrate.^18,19^ Regardless of these throughput gains, MSI molecular image generation is still a lengthy process that requires the implementation of postacquisition image analysis algorithms to extract phenotypic chemical information from the data sets.

In this study, we develop a single raster DESI-MSI line-scan strategy that reduces acquisition times by decreasing the amount of spatial information required for rapid biochemical analysis. This “Fast-Pass” workflow was first optimized on a linear array of samples consisting of multiple analyte classes analyzed under differing experimental conditions and acquisition parameters. The Fast-Pass approach was then applied to the untargeted phenotyping of microorganisms through the evaluation of mutant *E. coli* strains that had been genetically engineered to produce different levels of various target FFAs. Distinct production profiles were observed in each sample that directly correlate to the types of thioesterase modifications present in the engineered strain from which it was derived. While FFA producing *E. coli* serves as a model system, the Fast-Pass workflow performs with the necessary analytical attributes to be applied to diverse biological systems and can be directly integrated into established untargeted metabolomic and lipidomic workflows. Ultimately, the optimized DESI-MSI Fast-Pass workflow was able to provide comparative production levels of the target bioproducts of interest as well the global metabolic profiles of individual strains on a subminute time scale, which is approximately an order of magnitude faster than previous DESI screening results.

## Methods

### Standards and Chemicals

Peptides (VTV, bradykinin, angiotensin II, substance P, and leucine enkephalin), carbohydrates (maltobiose, M2; maltotriose, M3; maltotetraose, M4; maltopentaose, M5; and maltohexaose, M6), and exogenous compound standards (caffeine, sulfadimethoxine, sulfaguanidine, and reserpine) were purchased from Sigma-Aldrich. Fatty acid standards (octanoic acid, C8:0; decanoic acid, C10:0; dodecanoic acid, C12:0; and tetradecanoic acid, C14:0) were purchased from Cayman Chemical Company. Sodium acetate as well as high-purity (Optima-grade) methanol, water, and acetonitrile were obtained from Fisher Scientific. A mixture of 14 heavy-labeled lipids (SPLASH LIPIDOMIX) was purchased from Avanti Polar Lipids. All chemicals were used as received.

### Strains and Culture Conditions

*E. coli* strains TY05, NHL17, and TY04 were designed and engineered to produce various chain lengths of FFAs at differing production levels under isopropyl-d-thiogalactopyranoside (IPTG) induction.^20,21^ Namely, TY05 was designed to preferentially produce C12:0, NHL17 produces C8:0, and TY04 acts as a negative control strain (Table S1). For DESI-MSI analysis, 5 mL cultures of each strain were inoculated from freezer stocks and incubated overnight in LB broth at 37 °C with shaking. 50 mL cultures of each strain were then grown to an initial optical density at 600 nm (OD_600_) of 0.3, incubated for two h at 37 °C with shaking, then induced with 50 μL of 1 M IPTG. Finally, the cultures were allowed to grow for 24 h at 30 °C with shaking after IPTG induction to assess FFA production. The liquid cultures were flash frozen prior to preparation for DESI-MSI analysis (Figure S1).

### Sample Preparation

For the initial investigation of the Fast-Pass workflow, standard solutions representing four different analyte classes (peptides, exogenous compounds, carbohydrates, and fatty acids) were prepared in separate equimolar mixtures at 50 μM in 50/50 methanol/water. Equal concentration of sodium acetate (50 μM v/v) was added to the maltose sugar mixture to promote ionization. Fast-Pass analysis was carried out on 10 μL aliquots of the standard mixes deposited directly onto a 4 × 11 grid of raised polytetrafluoroethylene (PTFE) spots (Figure S2) and allowed to evaporate to dryness (∼30 min). Each PTFE spot is 3 mm in diameter, and each row contains 11 spots spanning 50 mm in edge-to-edge total length. Mixtures of peptide hormones, exogenous compounds, and carbohydrates were all analyzed in positive ionization mode, whereas fatty acid analysis was conducted in negative ionization mode.

To evaluate Fast-Pass experiments with the *E. coli* strains, frozen liquid cultures corresponding to three biological replicates of each strain were thawed and their lipophilic compounds were extracted using a modified liquid–liquid extraction (LLE) procedure.^22^ Briefly, the samples were normalized based on their OD_600_ values, subjected to an overnight protein crash in methanol at −80 °C, and then extracted via LLE implementing methyl *tert*-butyl ether (MTBE):methanol/water. During LLE, exogenous lipids and heavy labeled standards were added to each sample for quality control purposes. The resulting hydrophobic layer was vacuum concentrated, then reconstituted in 90/10 methanol/chloroform, and deposited onto the sample slide PTFE spot array in a pseudorandomized pattern. In addition to the nine strain samples (three biological replicates of three strains), an LB medium sample was processed simultaneously and spotted for use as a matrix blank during data analysis. For these experiments, the 4 × 11 grid of PTFE spots was replaced by a 3 × 10 grid of uncoated wells masked by PTFE (Figure S2), which allows the low surface tension methanol/chloroform mixture to be spotted without modifying the composition and subsequently decreased the time associated with evaporation to less than 5 min. A media blank subtraction was applied to the integrated FFA signals prior to comparison to ensure that the signal profiles originated from the engineered strains rather than the exogenous media. These FFA signals were then normalized on an analyte-by-analyte basis and subsequently plotted for comparison across the samples.

### DESI Source Parameters and Data Processing

Experiments were carried out on a Waters Synapt G2-S high definition mass spectrometer (IM-MS, Waters Corporation) fitted with a prototype DESI-MSI probe designed by Tillner et al.^23^ Optimized source parameters were found to be as follows: ±5 kV capillary voltage, 150 °C source temperature, 0.45 bar of N_2_ gas flow, a 70° sprayer angle, a 0.7 mm ablation diameter, and an *x*, *y*, *z* positional orientation relative to the MS inlet of −2, +2, +2.75, respectively. The DESI ionization solvent consisted of 90/10 acetonitrile/water, 0.2 ng/μL leucine enkephalin, and either 0.1% formic acid for positive ion mode or 0.1% ammonium hydroxide for negative ion mode. Pixel dimensions for Fast-Pass experiments were 50 μm × 1000 μm. The IM-MS was mass calibrated with sodium formate salt clusters to a 95% confidence band. All acquisitions were performed in triplicate in the mass range of *m*/*z* 50–1200.

Raw data files were initially processed in HDImaging, with the measured intensities of the 500 most abundant features being prioritized for statistical screening. Lock mass adjustments were performed using leucine enkephalin present in the spray solution and processed *.txt files were imported into Microsoft Excel for further data treatment. Imported data were spatially normalized to the leucine enkephalin signal to adjust for ionization, sample, and spray inconsistencies over the course of the raster. Tentative analyte identifications were made by searching measured *m*/*z* values against the *E. coli* Metabolome Database (ECMDB), Kyoto Encyclopedia of Genes and Genomes (KEGG), and ChemSpider, with annotations based on a mass measurement threshold of 5 ppm and 80% isotopic envelope similarity.

## Results and Discussion

### Fast-Pass Methodology

**Figure 1:**
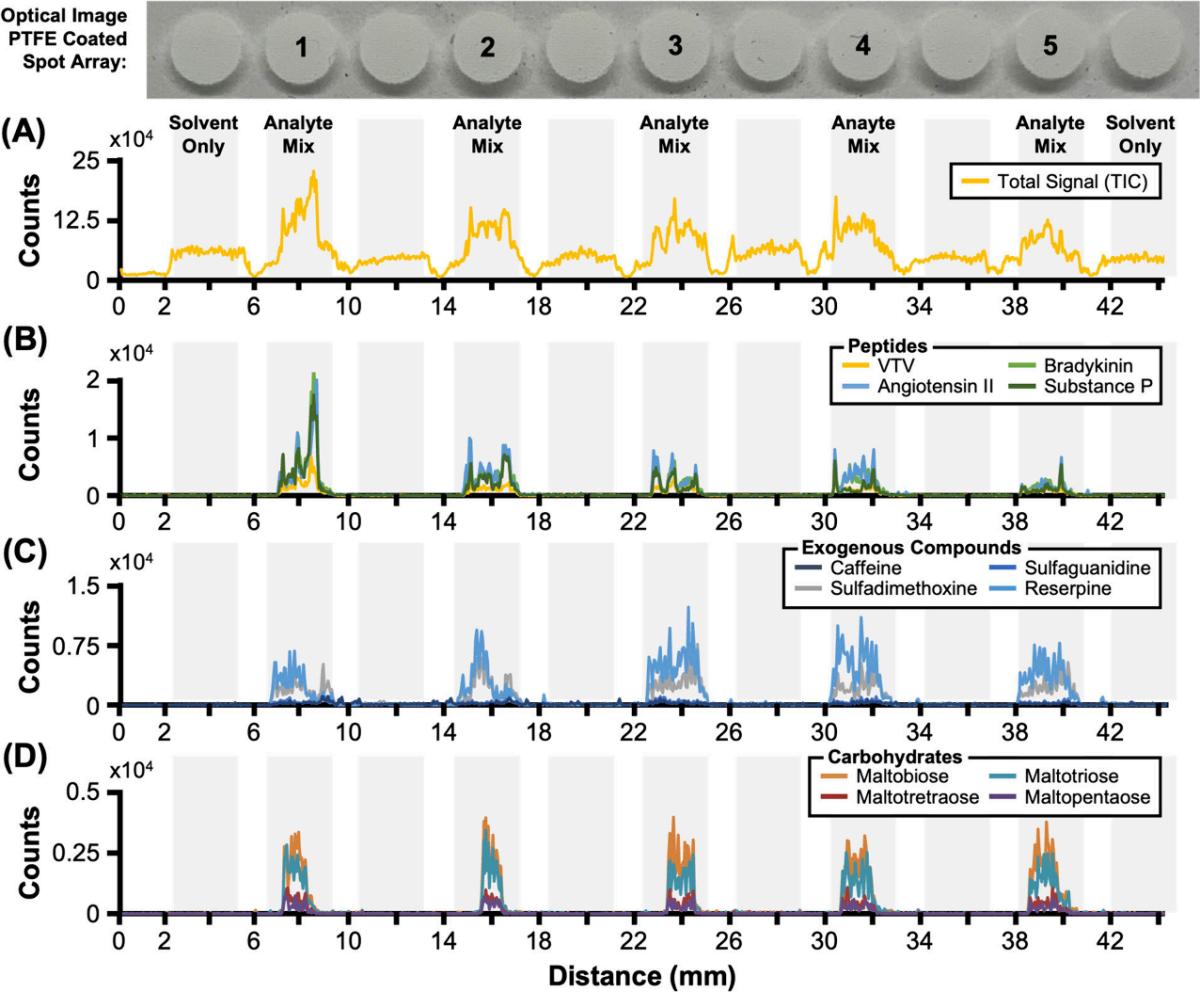
(A) Fast-Pass line-scan experiments are projected as a total ion chromatogram (TIC) in which each signal frame corresponds to a distance along the linear array of samples. Extracted ion chromatograms (XICs) for peptide hormone mix (B), exogenous compounds (C), and carbohydrates (D) emphasize the location-resolved nature of the acquisitions. Analyte signal is largely conserved within the sample bounds of the individual spots.

The time-consuming aspect of established DESI-MSI workflows is the row-by-row rastering required to produce spatially resolved, 2D ion images. In practice, a significant fraction of the total MSI acquisition time is spent on relocating the DESI probe to the next row in the sample scan. These long-format acquisitions can require tens of hours for image generation and frequently utilize computationally intensive image analysis algorithms.^24,25^ In this work, linear location information provides reproducibility measurement, rather than spatial imaging information within an area measurement; thus, a line-scan is “fit-for-purpose” to balance the need for rapid measurement with the information content provided by the measurement. By depositing samples onto predefined locations within a linear array (Figure 1A), the same multiplexed analysis can be achieved through a single line-scan, with analyte locations predetermined by the spotting pattern.^26−28^ This approach removes sample location as a variable and foregoes the need for multiraster acquisitions, increasing throughput and dramatically decreasing the time frame associated with individual experiments. The resulting line-scan data can be projected in a manner similar to that of an LC chromatogram, wherein the retention time dimension is replaced with a distance dimension, as the spatial location of each sample spot is fixed, and thus known (Figure 1A). Integrating the signal across preestablished distances provides full MS spectral information for each sample spot within the linear array, allowing for reproducible, frame-exact comparisons across the different sample locations.

In order to evaluate the Fast-Pass method’s application to diverse sample types and gauge performance in differing modalities, standard mixtures comprised of various analyte classes were deposited, allowed to dry, and then analyzed. Isolating the traces for single *m*/*z* values measured during an acquisition provides analyte-specific readouts that underscore the location-resolved nature of the Fast-Pass approach (Figure 1B). Here, each peptide hormone was detected in high abundance and localized within designated analyte spots with negligible carryover to adjacent sample locations. Because all analyte classes were prepared in equimolar mixtures, variation in measured signal intensities corresponds primarily to ionization differences associated with the specific analyte as well as differential signal partitioning into multiple ion channels (e.g., [M + H]^+^, [M + 2H]^2+^, adduct coordination, etc.).

Two notable observations can be made from the traces in Figure 1: (1) the localization of signal within anticipated sample boundaries was observed for each analyte investigated and (2) signal response profiles for the same analyte classes within each sample spot are highly correlated. Signal localization within each spot indicates specific DESI sampling with minimal sample carryover, whereas the similar response profiles for each compound class suggest these are partitioning in distinct locations within each sample spot during droplet drying. Specifically, peptide signal is more abundant on the spot margins (sometimes referred to as the “coffee ring” effect), whereas the exogenous compounds distribute more evenly across the spot area, and the carbohydrates tend to concentrate at the center of each dried droplet, which is expected based on hydropathy considerations. However, heterogeneous spatial distributions are still observed as a result of the evaporating process taking place under ambient conditions.^29,30^ Similar traces were observed for standard mixtures of FFAs measured in negative polarity with these traces serving as the basis for subsequent raster rate analyses. Despite these intraspot variations, comparisons of the integrated analyte signal from one spot to another were found to be highly reproducible, with percent covariances of less than 10% on average (Table S2).

### Raster Rate Optimization

**Figure 2:**
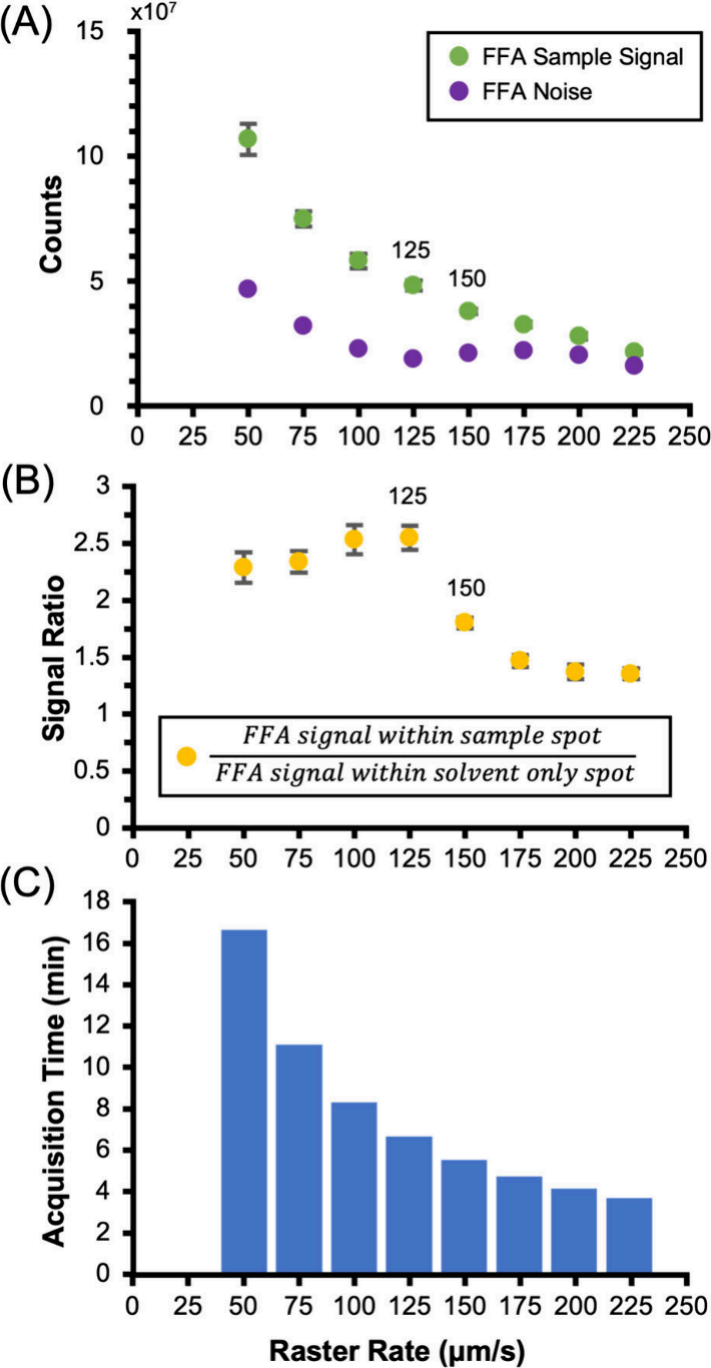
(A) Inverse relationship between FFA sample signal and raster rate is exhibited in green. Abundance of FFA noise projected in purple, representing signal observed in blank solvent spots. (B) Signal ratio of spotted FFAs to blank solvent spots corresponds to the comparison of FFA signal measured within a sample spot to FFA signal detected within a solvent only spot at a specific raster rate. Individual data points are the average across six spot replicates (*n* = 6). (C) A plot of acquisition time associated with each raster rate over a distance of 55 mm.

One of the defining parameters of the Fast-Pass workflow is the raster rate associated with the single line scan or the rate at which the sample stage moves under the stationary DESI source during an acquisition. Importantly, the raster rate does not affect the number of pixels recorded during an acquisition, as that is solely determined by the distance of the line-scan being imaged and the data acquisition pixel size. However, because DESI is a continuously ablative technique, raster rate does affect spectral scan time, which is the amount of time spent collecting signal for a particular pixel frame. At higher raster rates and shorter scan times, it would be anticipated that the measured signal within each pixel would decrease. While maximizing throughput was the primary objective during the development of the workflow, it was important to balance increased speed with retaining high levels of chemical signal and limiting the amount of ablation-related sample delocalization. To determine optimal scan speeds, signal ratios for spotted mixtures of FFA standards were evaluated at various raster rates (Figure 2A).

Signal ratios were calculated by comparing the analyte signal integrated across sample spots with the noise evaluated between the sample spots (Figure 2B), with the latter being considered a convolution of background noise at the same *m*/*z* and delocalized analyte signal. A mixture of FFAs was used for raster rate optimization, as this class comprises the main bioproducts of interest for the engineered *E. coli* strains under investigation. Eight different raster rates were evaluated, corresponding to acquisition timeframes ranging from 3.70 to 16.67 min over a row length distance of 50 mm (Figure 2C). During optimization experiments, signal data were averaged across six spot replicates.

The anticipated relationship between faster raster rates and decreased measured signals was observed. However, the same potential relationship between the raster rate and the delocalized signal was not observed within the bounds of solvent only spots. Specifically, a 60% decrease in the normalized intensity of delocalized signal occurred from 50 μm/s to 125 μm/s, but for 150 μm/s and faster, this signal was relatively constant. This differential response is likely the result of the sample delocalization inherently associated with DESI ablation and the suboptimal flow dynamics of ion transfer for location-resolved studies at higher raster rates. Because these effects combine with a reduction in signal due to shorter scan times, a maximum signal ratio occurs for FFA experiments acquired at 125 μm/s, which was subsequently identified as the optimal raster rate for Fast-Pass acquisitions. Similar raster rate analyses utilizing the other compound classes (peptide hormones and exogenous compounds) were found to be in agreement with the rate determined via FFA analysis (Figure S3). As such, the raster rate for all line-scan experiments, including those for untargeted molecular phenotyping on biological systems, was performed at 125 μm/s (7.5 mm/min). This marks a throughput of 40 s per sample for a 55 mm, 11-sample line-scan with acquisition time of 440 s (Figure 2C).

### Fast-Pass for Bioanalytical Screening

**Figure 3:**
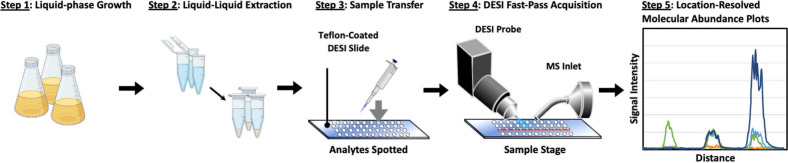
Flow diagram depicting the DESI Fast-Pass workflow. Strains are grown in liquid culture in step 1 before undergoing an MTBE-based LLE in step 2, wherein internal standards are added to the samples for quality control purposes. Analytes are then spotted directly onto a DESI slide and allowed to evaporate to dryness under ambient conditions.

In order to evaluate the Fast-Pass workflow on biological samples, three strains of genetically engineered *E. coli* were evaluated, two IPTG inducible strains (TY05 and NHL17) and one negative control strain (TY04). TY05 is expected to produce primarily C12:0 due to the insertion of multiple copies of a codon-optimized acyl–acyl carrier protein, thioesterase, while NHL17 was engineered to predominantly produce C8:0 through the same mechanism but with only a single copy. The negative control strain, TY04, contains two catalytically inactive versions of the thioesterase modification present in TY05.^20,21^ The full Fast-Pass strain screening workflow is summarized in Figure 3, with the samples grown as liquid cultures, extracted via LLE, manually spotted onto glass slides, analyzed through Fast-Pass DESI-MSI, and interpreted as location-resolved molecular abundance plots.

**Figure 4:**
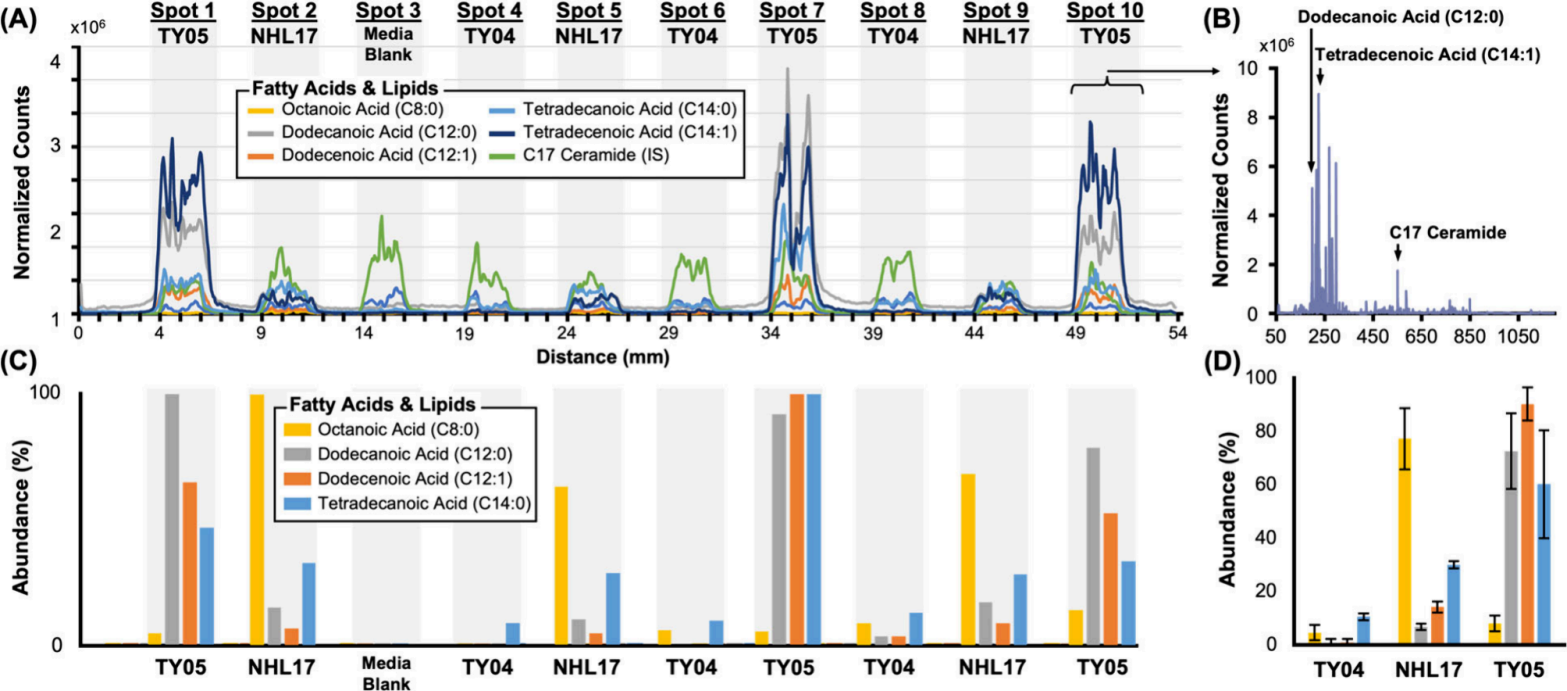
(A) XIC traces for the selected *m*/*z* values. Green trace corresponds to an internal standard added during LLE, while all other traces are various FFAs. (B) Mass spectra projection of signal integrated across frames corresponding to sample location “Spot 10”. Notable analytes are annotated within the spectrum including various bioproducts of interest and internal standard peaks. (C) FFA abundances for each spotted sample, with the signal having been blank-subtracted and normalized on an analyte-by-analyte basis. Spot 3 corresponds to media matrix blank. (D) Relative, blank-subtracted titer comparison between strains emphasizes varied levels of FFA production.

The Fast-Pass approach was able to characterize the FFA production of all three strains while simultaneously providing untargeted global metabolic information for each sample. The location and abundance of various FFAs are highlighted by isolating the measured signal associated with individual *m*/*z* values (Figure 4A), indicating that the C12:0 signal is primarily confined to TY05 sample locations while C8:0 is correlated to NHL17 locations, both as anticipated. Fast-Pass was also able to detect minor FFA products, such as the monounsaturated fatty acid dodecenoic acid (C12:1) in TY05--a known side product of the thioesterase used.^31^ However, other chemical species, such as C14:0, appear to have more independent correlations to the target products of TY05. The presence of tetradecenoic acid (C14:1), found in both engineered strains but measured in particularly high quantities in TY05 locations, represents similar side product detection as was seen for C12:1. Some lipid species displayed no correlation to the engineering strategies present, for example PG 36:2 was found in all three strains, including the TY04 negative control strain, and at similar production levels (Figure S4). Integrating all signals corresponding to specific sample locations provides comprehensive MS spectral information for each individual strain within the linear array (Figure 4B). These integrated spectra allow for the reproducible, frame-exact comparisons to be used for subsequent untargeted multivariate analysis.

Exogenous lipids and heavy labeled quality control standards were added to every sample, including the media blank, during sample preparation as a means to measure instrument and process variability, respectively. The signal for one such exogenous compound (C17 ceramide) is visualized by the green-trace-extracted ion chromatogram (XIC) in Figure 4A, where it is present in all sample locations and serves as the primary peak associated with both the media blank and the negative control samples. On average, the percent covariance (%CV) values for these variability measurements were less than 20% across all samples and blanks, with the %CV being less than 10% on average for biological replicates of the same strain. This indicates, in part, that the Fast-Pass workflow outlined can function reproducibly as an ambient bioanalytical screening tool (Table S2).

The FFA production for each of the sample profiles (Figure 4C) provides a direct readout of the effect that the genetic edits have on bacterial metabolism. While the XICs emphasize the spatial nature of the Fast-Pass MSI method, these production profiles are particularly useful for qualitative comparisons of product yields. Aggregate FFA production titers (Figure 4D) provide more wholistic strain comparisons while also illustrating that these differences fall outside of the error of repeatability associated with biological replicates. The application of this Fast-Pass workflow to profile comparisons was found to be repeatable through interday replicates, with the same FFA expression patterns and titer observations being conserved during subsequent analyses conducted on different days and at different times of day (Figure S5).

**Figure 5:**
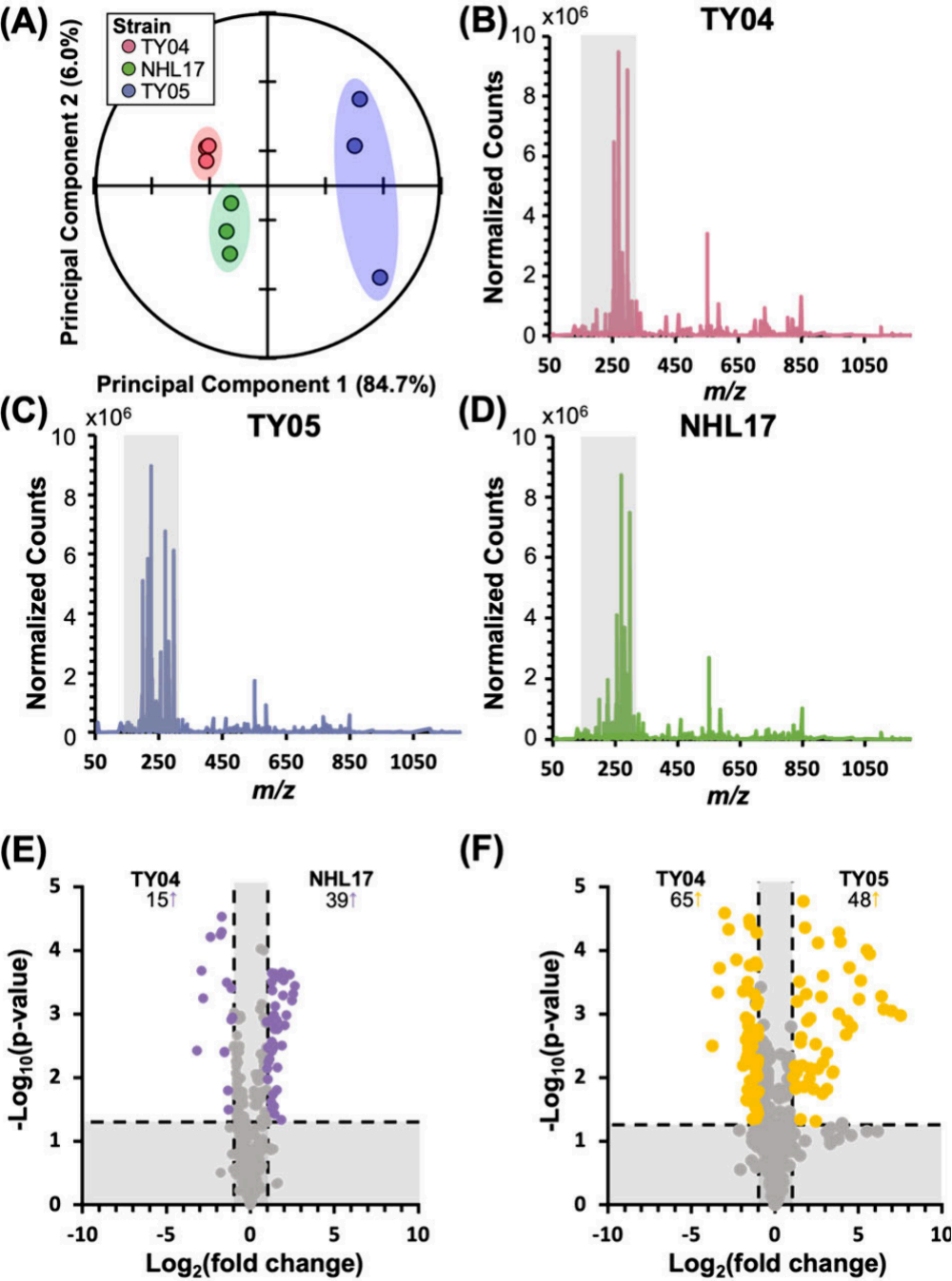
(A) PCA identifies three distinct phenotypes within the nine bacteria samples. (B–D) Example mass spectra for each of the three strains. Typical mass range of free fatty acids designated by shaded region. (E, F) Volcano plots identify both upregulated and downregulated features for the two engineered strains as compared to the negative control.

Full, global chemical profiles for each strain were generated by integrating all signals present within sample distances for each of the 9 sample locations (Figure 5). These sample profiles were treated as broad scale MS acquisitions and evaluated through principal component analysis (PCA) to assess molecular differences (Figure 5A). Three distinct phenotypic groupings were identified among the 9 samples, demonstrating overall metabolic distinctions across samples of different strains as well as grouping of biological replicates within strains. Investigating the loading data supports the information obtained from both XICs and FFA production profiles. For example, C12:0 is one of the primary features of principal component 1 (PC1), contributing to the separation observed between TY05 and the other two strains (Figure S6). C14:1 also contributes to the separation observed from PC1, validating the visual information provided by the location-resolved XICs and providing an additional means of elucidating off-target production and product sinks.

Differential comparative analysis of the untargeted data across different strains allows for a potentially deeper understanding of off-target effects that specific genetic edits are having on bacterial metabolism. The statistical significance of features identified within the biological replicates of both NHL17 (Figure 5E) and TY05 (Figure 5F) was evaluated using volcano plot projections, with the negative control strain, TY04, serving as the basis of comparison. When evaluating the total number of significant features (*p* ≤ 0.05; |fold change| ≥ 2) in the two comparisons, more features with overall significance are identified in the TY05 compared to TY04 than in NHL17 compared to TY04, with 113 and 54 significant features identified, respectively. This corroborates the sample clustering of the phenotypic information observed in PCA, with spacing between both PC1 and PC2 occurring between biological replicates of TY04 and NHL17 being closer than those of TY05. Additionally, of the 113 significant features identified in the TY05 comparison, 48 of those features are upregulated (fold change ≥ 2) in comparison to TY04. The remaining 65 features are downregulated (fold change ≤ −2) in comparison to TY04. Many of the significant features observed as upregulated in TY05 correspond to the desired FFA products directly engineered and other products accumulated as a result of the corresponding genetic edits. The notable difference in the number of significant features identified in each comparison could indicate variation in how extensively the bacterial metabolome is modified by the two different engineering strategies used in the respective strains. Importantly, it emphasizes that off-target modification of the metabolome beyond targeted products is an anticipated consequence of genetic editing and should be monitored and evaluated in strain optimization. An annotated feature list for all untargeted metabolic data is presented in Table S3, while the significant features identified by the volcano plots are outlined in Table S4 and Table S5.

## Conclusions

We demonstrate that a Fast-Pass DESI-MSI workflow is an effective high throughput alternative to comprehensive imaging MS for the rapid bioanalytical screening of genetically engineered microbes. The single raster strategy was found to significantly reduce the acquisition time scale by limiting the spatial information required for sample differentiation while retaining the full spectral information on the untargeted analysis. Because this approach analyzes a linear array of samples deposited at defined locations, sample indices are predetermined; thus, no image analysis algorithm is required to dictate regions of interest, streamlining subsequent data processing and interrogation. The Fast-Pass workflow was applied to various analyte classes in multiple modalities and, in each case, was able to provide reproducible, location-resolved chemical profiles on the subminute time scale. The Fast-Pass analysis of genetically engineered *E. coli* strains identified distinct FFA production profiles between the samples that correspond directly to the types of thioesterase modifications present in each engineered strain. Through analysis with the Fast-Pass workflow, TY05 and NHL17 were accurately identified as the predominant producers of C12:0 and C8:0, respectively. Differential metabolomics of the untargeted data demonstrated overall metabolic distinctions across strains (PCA) as well as information regarding the significance of individual features (volcano plots).

This workflow represents an improvement in the time frame associated with bioanalytical screening of genetically engineered microbes by more than an order of magnitude, reliably measuring small molecules and providing global metabolic information for a strain in ∼40 s. This work addresses a major bottleneck in the analysis of synthetic biology strategies and moves us closer to parity between the speed at which metabolic engineers can produce edits and the speed at which analytical chemists can characterize the effects of those edits. While current identifications are based on mass measurements alone, future work will include the implementation of tandem MS fragmentation strategies in order to increase the confidence of molecular annotation. Additionally, DESI-MSI has demonstrated the effectiveness in analyzing a diverse array of biological systems. As such, Fast-Pass is not limited to the investigation of FFA production in *E. coli*.^32−34^ Integration of the Fast-Pass methodology as a true screening step in the deep metabolomic investigation of genetically engineered systems will help to inform resource management with regard to more time-consuming multidimensional MS analyses, specifically those that seek to obtain high levels of information such as ion mobility (IM) integrated LC-IM-MS/MS.^35^ Additionally, because the same extracts analyzed through Fast-Pass are amenable to those deeper analyses (e.g., LC-IM-MS and LC-IM-MS/MS), Fast-Pass data can be integrated into established untargeted metabolomic and lipidomic workflows.

## Supporting Information Available

Strain genotype descriptions; in depth growth conditions and sample preparation workflows; photographs of sample slides for Fast-Pass analysis; selected percent covariances; selected XIC of strain correlated and uncorrelated features; loadings plot information used to derive PCA; XICs of Fast-Pass technical replicates; feature list of top 500 signals detected for *E. coli* during Fast-Pass with tentative identifications where applicable (PDF)

## Abbreviations

DESI-MSI: desorption electrospray ionization mass spectrometry imaging
ECMDB: *E. coli* Metabolome Database
FFAs: free fatty acids
GC: gas chromatography
IM: ion mobility
IPTG: isopropyl-d-thiogalactopyranoside
KEGG: Kyoto Encyclopedia of Genes and Genomes
LC: liquid chromatography
LLE: liquid–liquid extraction
MALDI: matrix assisted laser desorption ionization
MS: mass spectrometry
MTBE: methyl *tert*-butyl ether
*m*/*z*: mass-to-charge ratio
OD600: optical density 600
PCA: principal component analysis
PTFE: polytetrafluoroethylene
TIC: total ion chromatogram
XIC: extracted ion chromatogram
%CV: percent covariance
