# Supplementary material for: A Fast-Pass, Desorption Electrospray Ionization Mass Spectrometry Strategy for Untargeted Metabolic Phenotyping
Source: J Am Soc Mass Spectrom. 2025 Jan 27;36(2):400–8. doi: 10.1021/jasms.4c00459 (PMC11808754; doi:10.1021/jasms.4c00459)
Supplement: Supplementary file 1 — js4c00459_si_001.pdf [file js4c00459_si_001.pdf]

Supporting Information

A Fast-Pass, Desorption Electrospray Ionization-Mass Spectrometry Strategy for Untargeted Metabolic Phenotyping

Hawkins S. Shepard,[1] Jody C. May,[1] Baltazar E. Zuniga,[2] Joshua P. Abraham,[3] Brian F. Pfleger,[3] Jamey D. Young, [2] and John A. McLean[1]*

1. Department of Chemistry, Center for Innovative Technology, Vanderbilt University, Nashville, TN 37235
2. Department of Chemical and Biomolecular Engineering, Vanderbilt University, Nashville, TN 37235
3. Department of Chemical and Biological Engineering, University of Wisconsin, Madison, WI 53706

* Corresponding Author Email: john.a.mclean@vanderbilt.edu

SI Contents:

Figure S1 – In-depth sample preparation and growth conditions……….………………………S2
Figure S2 – Photographs of sample slides…………………………………………………… S3
Figure S3 – Raster rate analysis for peptides and exogenous compounds………………… S4
Figure S4 – Selected EICs …………………………………………………………………… S5
Figure S5 – FFA production profiles for replicates…………………………………………… S6
Figure S6 – Loadings plot for PCAs…………………………………………………………… S7
Table S1 – Strain descriptions………………………………………………………………… S8
Table S2 – Selected percent covariance for standards……………………………………… S9
Table S3 – Feature list for *E. coli* experiments……………………………………………… S10
Table S4 – Significant feature table for TY04 and NHL17 comparison……………………… S24
Table S5 – Significant feature table for TY04 and TY05 comparison………………………… S26

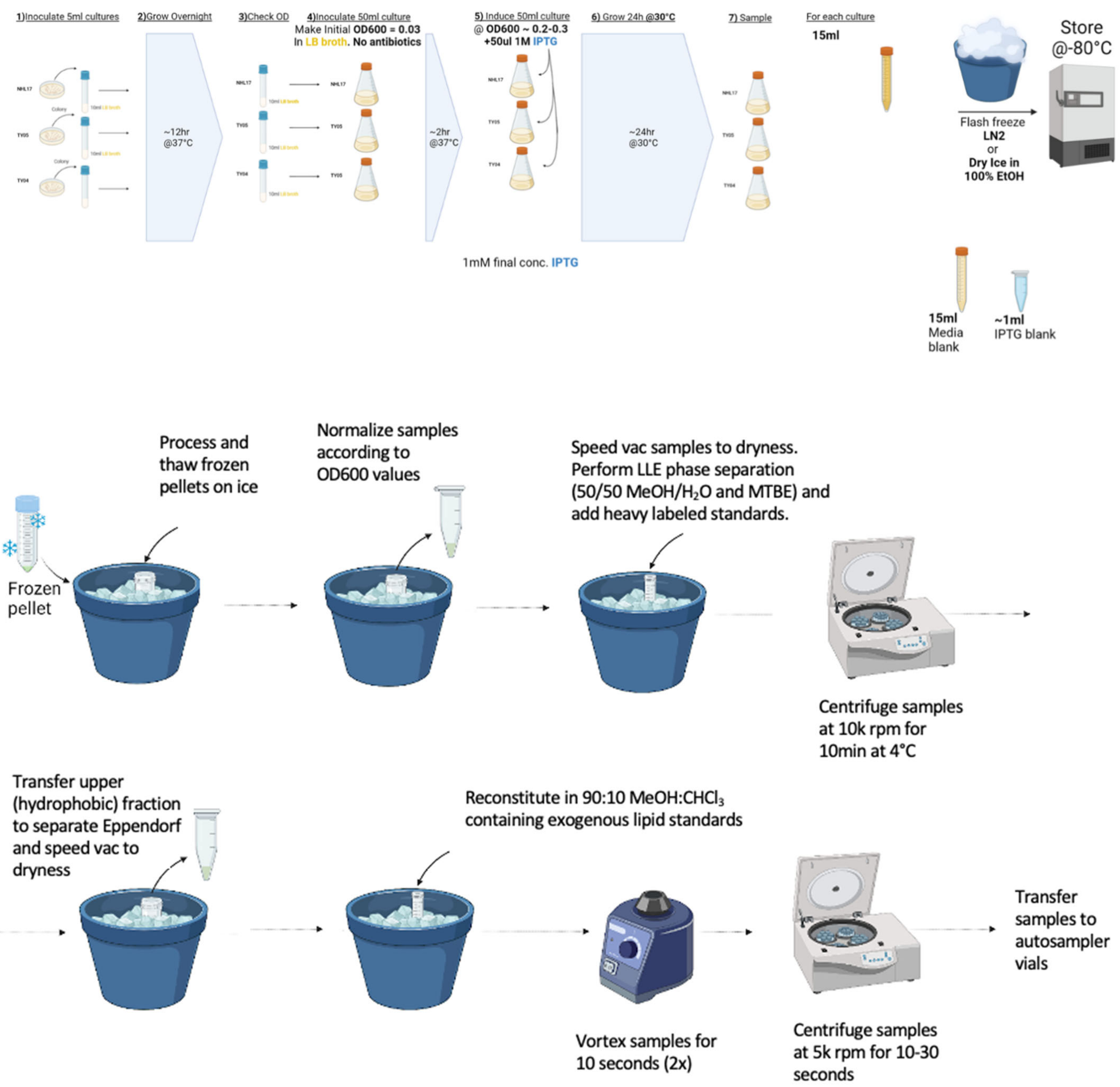

Figure S1: Sample preparation and growth conditions

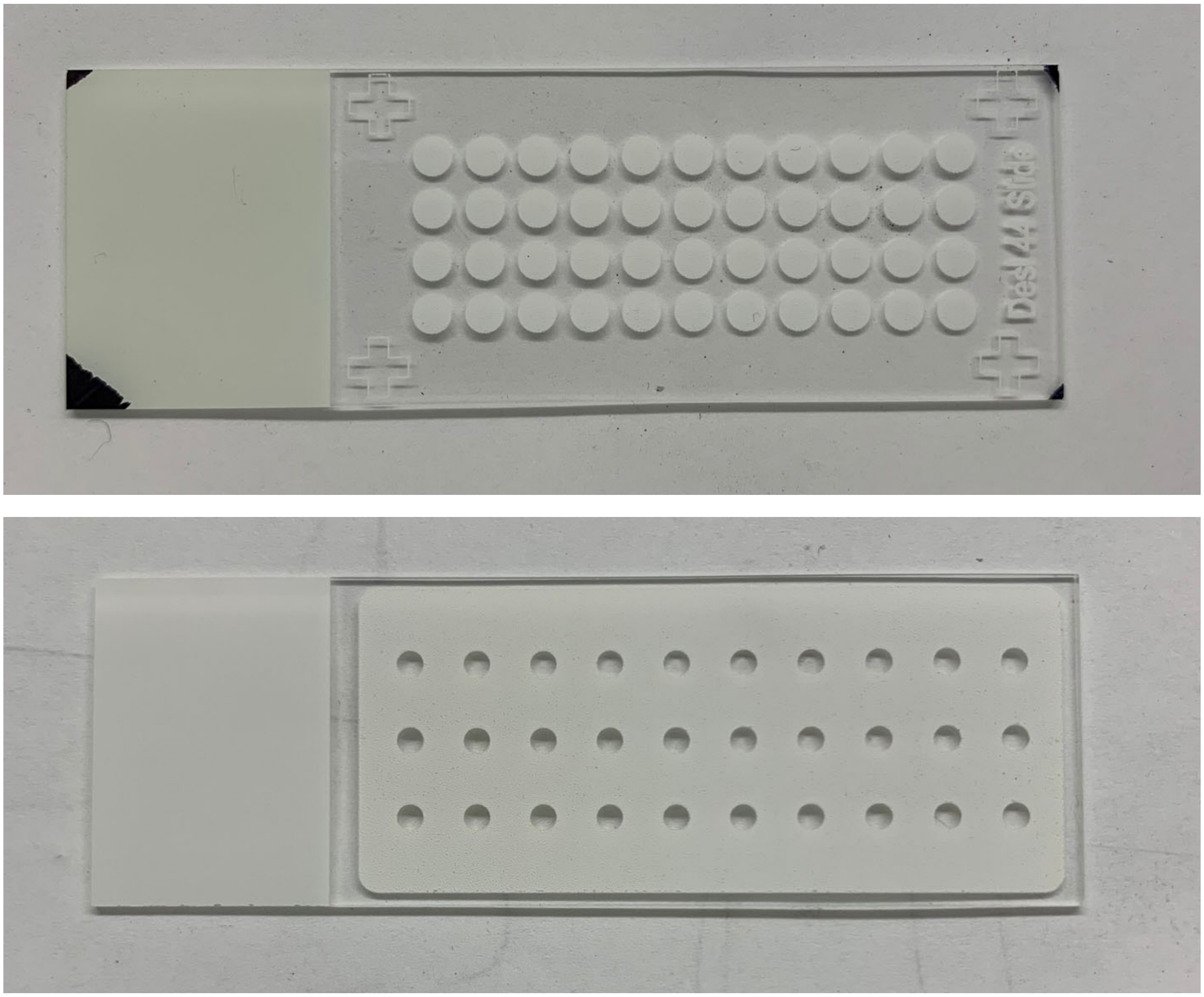

Figure S2: Optical images of PTFE-spotted and recessed well DESI sample slides

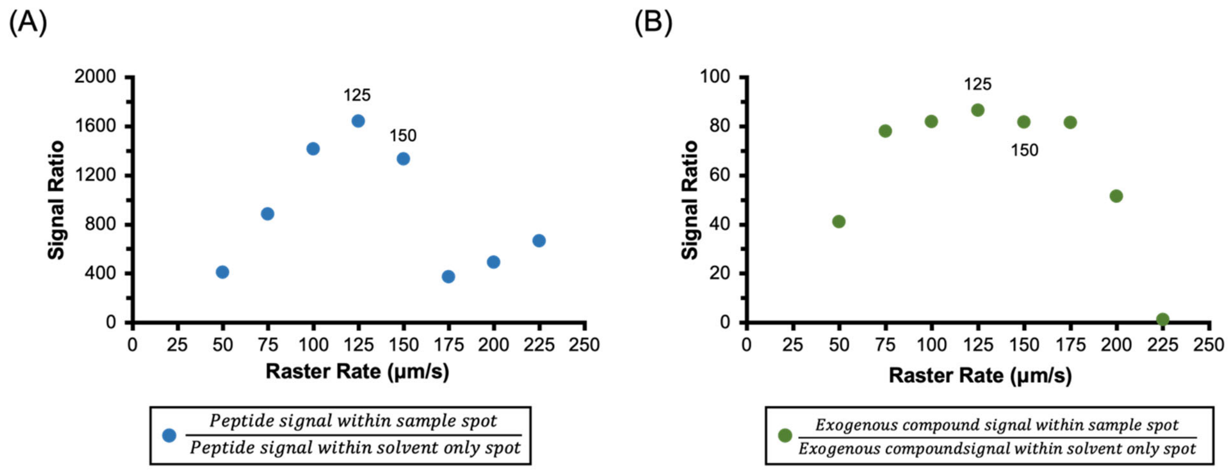

Figure S3: Signal ratio of spotted peptides **(A)** and exogenous compounds **(B)** at various raster rates. Plot data is averaged across six spot replicates (n=6).

(A) **C14:0 EIC**

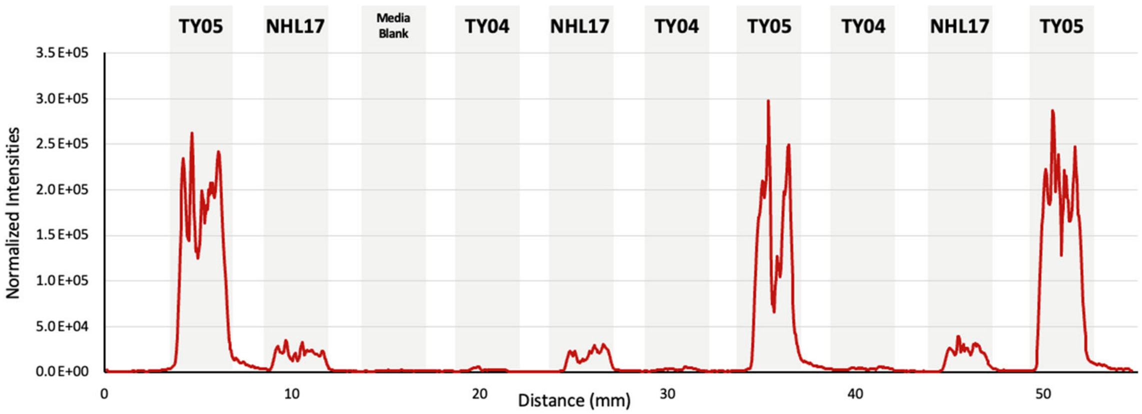

(B) **PG 36:2 EIC**

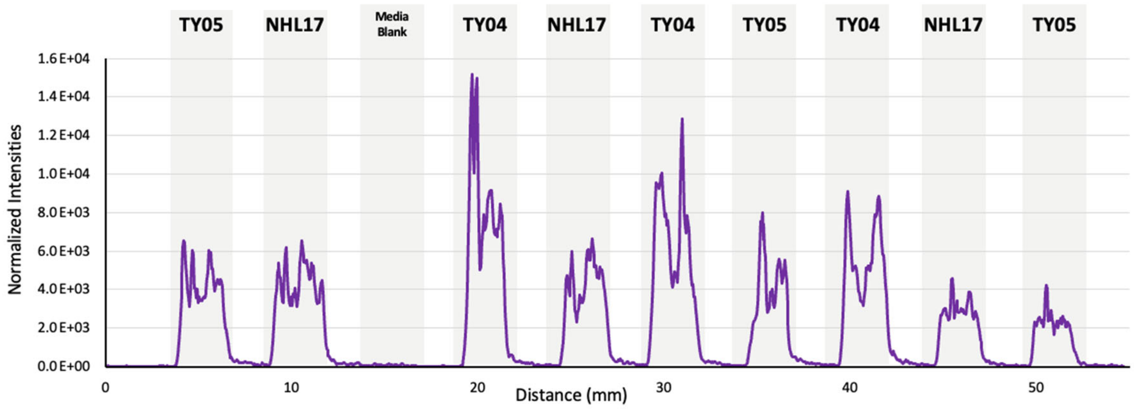

(C) **C19:0 EIC**

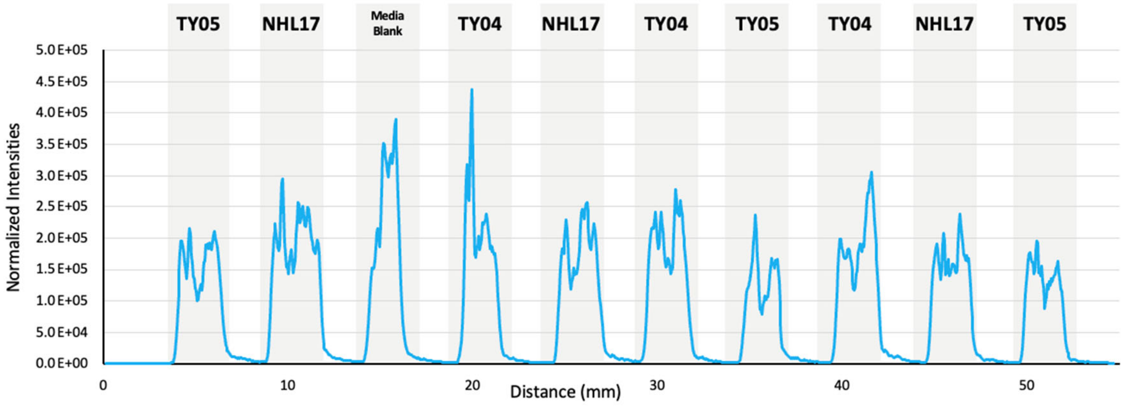

Figure S4: EICs of selected features representing both consistent and inconsistent signal response. Examples of inconsistent signal response include C14:0 **(A)** and PG 36:2 **(B)**, while consistent signal is exemplified by C19:0 **(C)**.

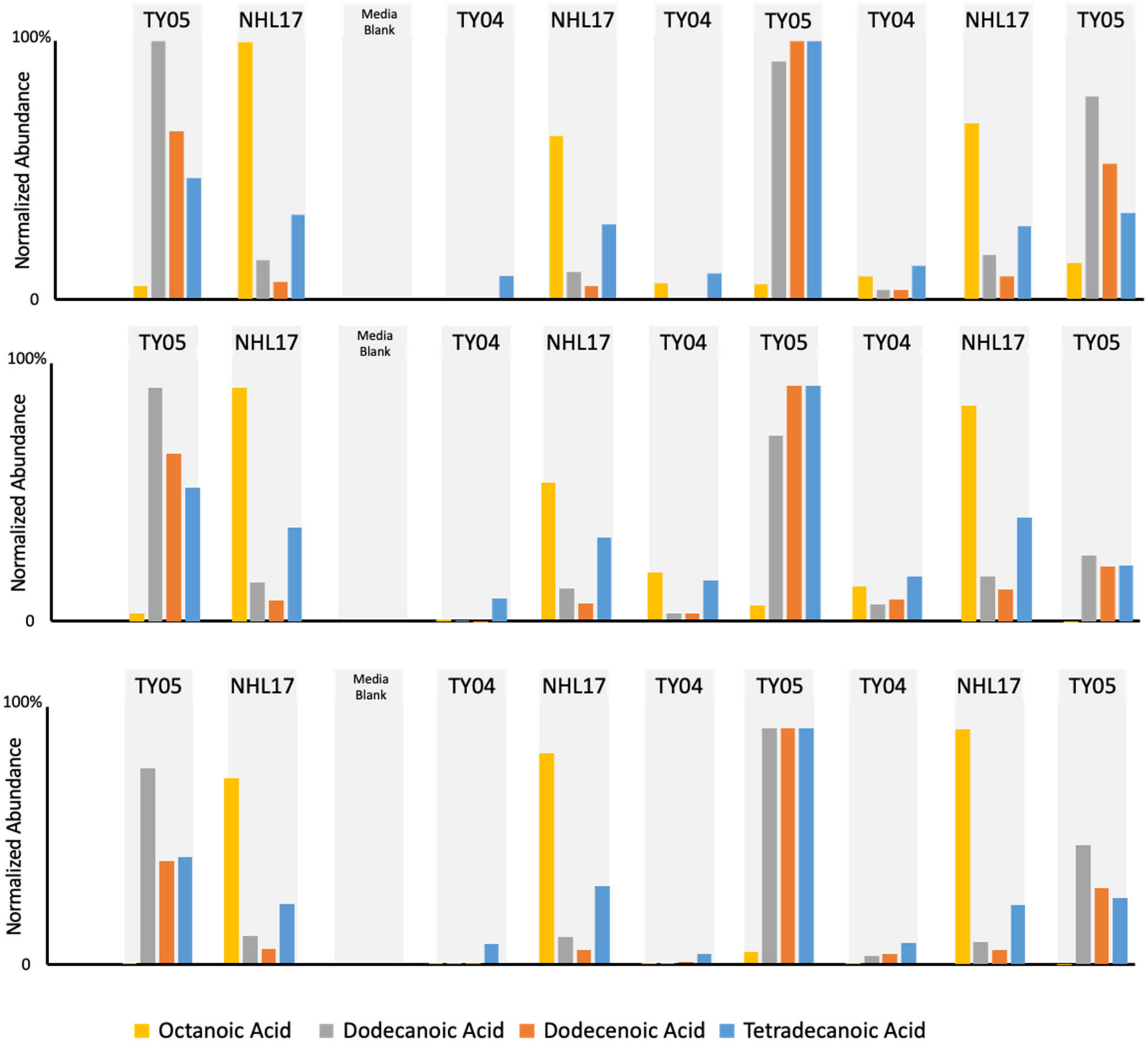

Figure S5: FFA production profile replicates. Each replicate was obtained on different days and at different times of each day.

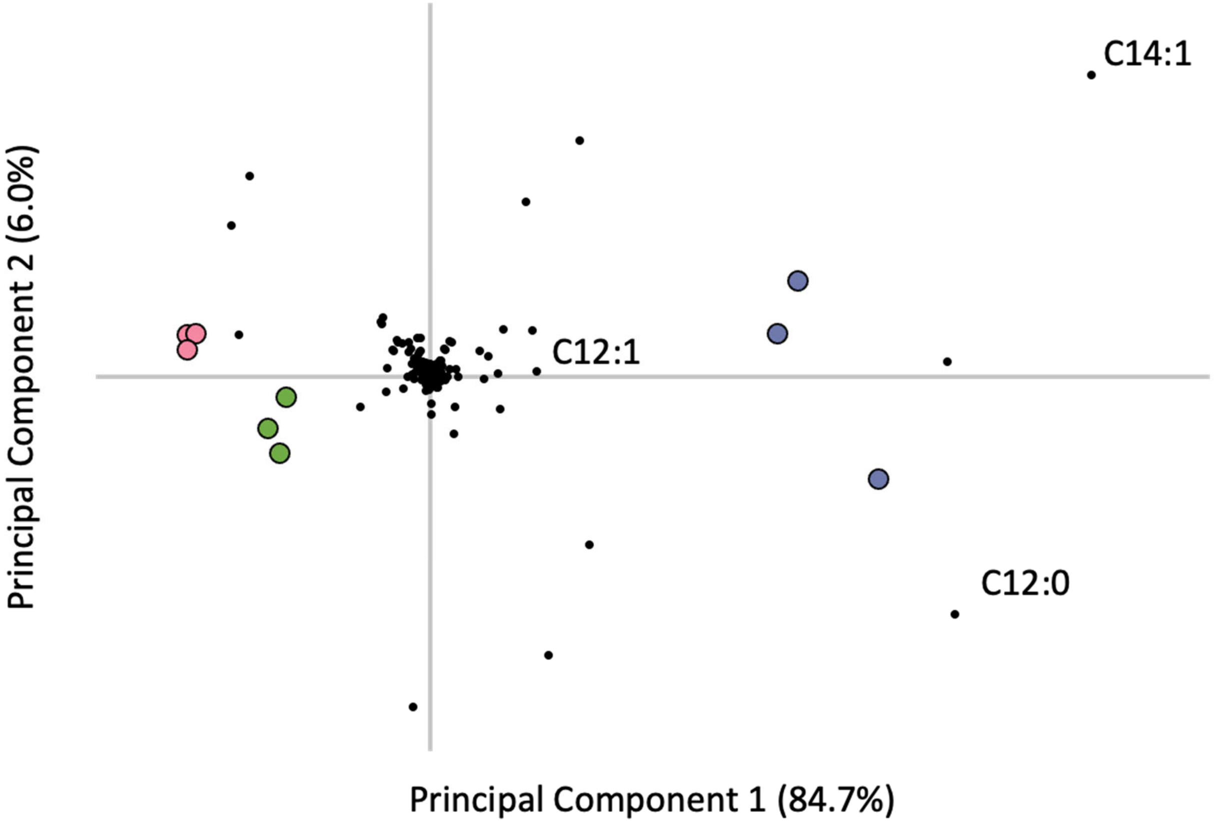

Figure S6: Loadings plot for PCA

Table S1: List of *E. coli* strains, genotype and primer used for their validation.

Strains	Genotype/construct	Primers	Reference
NHL17	K12 MG1655 ΔaraBAD ΔfadD::trc-CpFatB1.2-M4- 287 (C8-specific *Cuphea palustris* FatB1 thioesterase)	Forward (rNHL115) GCATCGTCCGTGGTAATCATTTG Reverse(rNHL93) GCATTTATGCCGATGTGAACGG	Hernández Lozada et al. 2018
TY05	K-12 MG1655 ΔfadDEAB::trcBTE (acyl-ACP thioesterase from *Umbellularia californica*)	Forward (fadABKO_colPCR_fwd) GGAGTGAATAAGTAACGCATCC Reverse (fadABKO_colPCR_rv) GCTGTCGCGTCTTATCGTGC	Youngquist et al. 2012
TY04	K-12 MG1655 ΔfadDE::trcBTEH204A (acyl-ACP thioesterase from *Umbellularia californica*)	Forward (fadEKO_colPCR_fwd) CGATTGATGGTAAAACGGTGTTGTT Reverse (fadEKO_colPCR_rv) CTGAAGTGCGGATAAAAACAGCAA	Youngquist et al. 2012

Table S2: Percent Covariances for relevant repeatability measurements.

Analyte	Ion Form	Covariance (%)*
Maltobiose	[M+Na]+	12.26
Maltotriose	[M+Na]+	9.30
Maltotetraose	[M+Na]+	11.02
Maltopentaose	[M+Na]+	3.65
Maltohexaose**	[M+Na]+	9.71
15:0-18:1(d7) PS	$[M-H]^-$	18.74 (19.80)
C17:0	$[M-H]^-$	17.65 (1.90)
C19:0	$[M-H]^-$	16.79 (9.44)
D-glucosyl-β1-1'-D-erythro-sphingosine	$[M-H]^-$	21.50 (8.19)
C17 Ceramide	$[M-H]^-$	22.90 (16.76)

*n=6 for Maltose sugar percent covariances; n=10 for internal standard percent covariances (parenthetical covariances are percentages calculated between biological replicates, n=3)

**Maltohexaose excluded from class specific EIC projection as it exhibits less than $5x10^2$ maximum counts per individual frame for sample locations

Table S3: Feature list table

m/z	Identification	Molecular Formula	Adduct	Mass Accuracy (ppm)	Total Abundance	Abundance Rank
57.3933	*	*	*	*	43067	451
59.0139	Acetic acid	C2H4O2	[M-H]-	< 0.1	721981	239
61.9884	Peroxynitrite	HNO3	[M-H]-	< 0.1	578374	329
67.1889	*	*	*	*	44274	491
73.3762	*	*	*	*	44537	487
94.9802	*	*	*	*	449412	425
98.9517	*	*	*	*	430257	355
116.9618	*	*	*	*	366742	478
122.9671	*	*	*	*	639842	294
130.0867	Leucine	C6H13NO2	[M-H]-	0.8	2165036	76
136.9364	*	*	*	*	1434507	123
138.9429	*	*	*	*	645541	247
143.1073	Octanoic Acid	C8H16O2	[M-H]-	0.7	908498	194
144.0451	*	*	*	*	1328440	136
147.0446	Phenyllactic acid	C9H10O3	[M-H2O-H]-	< 0.1	680246	232
151.0258	Xanthine	C5H4N4O2	[M-H]-	-2.0	1423996	115
154.9466	*	*	*	*	6257968	30
155.9466	*	*	*	*	710875	252
156.9474	*	*	*	*	871779	187
157.1228	*	*	*	*	1878684	97
159.0650	*	*	*	*	437591	409
161.0452	C6H12O6	C6H12O6	[M-H2O-H]-	1.2	612800	281
162.9597	*	*	*	*	1650873	103
164.0710	Phenylalanine	C9H11NO2	[M-H]-	-4.3	1876930	89
165.0599	*	*	*	*	256939	266
171.1022	*	*	*	*	648567	295
171.1386	Decanoic Acid	C10H19O2	[M-H]-	0.6	1950774	86

172.9575	*	*	*	*	1747731	106
174.0554	4-Hydroxy-5-phenyltetrahydro-1,3-oxazin-2-one	C10H11NO3	[M-H2O-H]-	-0.6	765278	227
176.9285	*	*	*	*	1798906	95
178.9339	*	*	*	*	480742	306
179.0600	*	*	*	*	243141	446
183.0095	*	*	*	*	371355	389
185.1539	*	*	*	*	844060	216
187.0974	Azelaic acid	C9H16O4	[M-H]-	-1.1	510475	334
188.9391	*	*	*	*	6787226	25
194.9396	*	*	*	*	925563	182
197.1541	*	*	*	*	4177491	41
198.1564	*	*	*	*	665933	255
198.9104	*	*	*	*	813595	191
199.0599	*	*	*	*	138970	393
199.1334	*	*	*	*	498880	344
199.1695	Dodecanoic acid (C12:0)	C12H23O2	[M-H]-	-1.5	23700139	7
200.1731	Dodecanoic acid (C12:0) C13	C12H23O2	[M-H]-	< 0.1	3150081	52
201.1759	*	*	*	*	325253	439
204.9133	*	*	*	*	850801	202
209.1541	*	*	*	*	916414	180
211.1693	*	*	*	*	740121	241
213.1490	*	*	*	*	1305226	124
213.1851	*	*	*	*	6999621	24
214.1893	*	*	*	*	1044292	150
215.1649	*	*	*	*	10591001	16
216.1684	*	*	*	*	1397181	122
216.9222	*	*	*	*	368199	401
220.8922	*	*	*	*	457388	340
222.9253	*	*	*	*	480564	327
224.9064	*	*	*	*	359385	431
225.1855	Tetradecenoic Acid (C14:1)	C14H25O2	[M-H]-	0.4	17691990	8
226.1890	Tetradecenoic Acid (C14:1) C13	C14H25O2	[M-H]-	1.3	2752775	59
227.2009	Tetradecanoic Acid (C14:0)	C14H27O2	[M-H]-	-0.9	12110773	12
228.2048	Tetradecanoic Acid (C14:0) C13	C14H27O2	[M-H]-	1.8	1901240	85
228.9312	*	*	*	*	3343431	49

229.1805	*	*	*	*	2260883	62
230.1842	*	*	*	*	360503	457
232.9241	*	*	*	*	1160622	131
233.1040	*	*	*	*	549071	300
233.1542	*	*	*	*	1305553	130
237.0799	*	*	*	*	496346	290
238.9020	*	*	*	*	777234	183
239.2011	*	*	*	*	1077912	146
241.1802	*	*	*	*	1845065	84
241.2168	Methylmyristic acid (C14:0;Me)	C15H30O2	[M-H]-	-2.1	4441455	32
242.1793	*	*	*	*	586322	270
242.2205	Methylmyristic acid (C14:0;Me) C13	C15H30O2	[M-H]-	-0.4	866178	163
243.1960	Hydroxymyristic acid (C14:0;OH)	C14H28O3	[M-H]-	-2.5	2668722	58
244.1993	Hydroxymyristic acid (C14:0;OH) C13	C14H28O3	[M-H]-	-2.5	432276	395
244.9074	4-Hydroxychlorothalonil	C8HCl3N2O	[M-H]-	-3.1	773086	169
246.8981	*	*	*	*	406542	386
248.0800	4-Nitro-7-piperazinobenzofurazan	C10H11N5O3	[M-H]-	4.4	818529	178
249.1426	*	*	*	*	332319	376
250.1443	2-Nitrophenyl octyl ether	C14H21NO3	[M-H]-	-2.0	476772	293
253.2164	Hexadecenoic acid (C16:1)	C16H29O2	[M-H]-	-3.6	13860174	10
254.2202	Hexadecenoic acid (C16:1) C13	C16H29O2	[M-H]-	-1.6	2458368	63
254.9061	*	*	*	*	464569	351
255.2323	Hexadecanoic acid (C16:0)	C16H31O2	[M-H]-	-0.4	40327263	3
256.2357	Hexadecanoic acid (C16:0) C13	C16H31O2	[M-H]-	< 0.1	7399388	23
256.9152	*	*	*	*	270577	460
257.2385	*	*	*	*	779430	158
259.1084	Alpha-ribosole (N1-(alpha-D-ribosyl)-5,6-dimethyl-benzimidazole)	C14H18N2O4	[M-H2O-H]-	0.5	880287	176
260.8813	*	*	*	*	288950	383
265.1472	*	*	*	*	3874222	42

266.1503	*	*	*	*	487813	275
267.1481	*	*	*	*	216749	430
267.2324	Avocadene	C17H34O3	[M-H2O-H]-	< 0.1	4464925	35
268.2360	*	*	*	*	857820	165
268.9240	*	*	*	*	683517	244
269.2119	*	*	*	*	881677	197
269.2479	Heptadecenoic acid (C17:0)*	C17H33O2	[M-H]-	-0.4	51454690	2
270.2510	Heptadecenoic acid (C17:0)* C13	C17H33O2	[M-H]-	-1.1	9690695	18
271.2471	*	*	*	*	597173	102
276.8883	*	*	*	*	237985	496
278.8950	*	*	*	*	518213	317
279.1631	*	*	*	*	545247	289
279.2324	*	*	*	*	532775	273
281.2477	Octadecenoic Acid (C18:1)	C18H33O2	[M-H]-	< 0.1	17017143	9
282.2511	Octadecenoic Acid (C18:1) C13	C18H33O2	[M-H]-	0.4	3434924	47
283.1619	*	*	*	*	262441	349
283.2635	Octadecanoic Acid (C18:0)	C18H35O2	[M-H]-	-0.7	25447912	5
284.2236	*	*	*	*	364008	402
284.2669	Octadecanoic Acid (C18:0) C13	C18H35O2	[M-H]-	-0.4	5073809	31
284.9005	*	*	*	*	470325	341
285.2698	*	*	*	*	588599	228
288.8868	*	*	*	*	293675	476
291.1601	*	*	*	*	311308	331
292.8907	*	*	*	*	228972	427
293.1060	*	*	*	*	316667	498
293.1783	*	*	*	*	2201783	68
294.1815	*	*	*	*	383587	423
294.9032	*	*	*	*	6131466	27
295.1686	*	*	*	*	105121	441
295.2260	*	*	*	*	513652	305
295.2635	Nonadecanoic Acid (C19:0)	C19H37O2	[M-H]-	-0.7	1555111	104
296.2665	Nonadecanoic Acid (C19:0) C13	C19H37O2	[M-H]-	-1.7	349566	429
297.1526	*	*	*	*	2275447	57

297.2454	*	*	*	*	1236730	137
297.2794	*	*	*	*	50664546	1
298.1562	*	*	*	*	461087	302
298.2827	*	*	*	*	9491681	13
298.9424	2-((2R,5Z)-2-Carboxy-4-methylthiazol-5(2H)-ylidene)ethyl phosphate	C7H7NO6PS	[M+Cl]-	-0.7	775963	207
299.2828	*	*	*	*	1358165	87
307.1933	*	*	*	*	371196	416
309.1727	*	*	*	*	568929	215
309.2791	*	*	*	*	409443	406
310.8775	*	*	*	*	1016140	144
311.1682	*	*	*	*	6906850	20
311.2947	*	*	*	*	1396971	108
312.1715	*	*	*	*	1334697	93
313.1671	*	*	*	*	439262	203
313.2385	*	*	*	*	348293	333
315.0672	*	*	*	*	383676	350
317.1461	*	*	*	*	222290	410
318.2063	*	*	*	*	610973	262
321.2096	*	*	*	*	321884	358
322.8876	*	*	*	*	269528	447
323.1839	*	*	*	*	172090	364
325.1837	*	*	*	*	6642583	21
325.3106	*	*	*	*	965446	120
326.1873	*	*	*	*	1690268	88
326.8568	*	*	*	*	245653	484
327.1808	*	*	*	*	438308	152
328.8897	*	*	*	*	315275	342
329.0816	*	*	*	*	431755	264
334.8958	*	*	*	*	3501922	34
337.2312	*	*	*	*	122187	193
338.8623	*	*	*	*	241068	475
339.1992	*	*	*	*	4284311	33
339.3265	*	*	*	*	3792630	40
340.2026	*	*	*	*	941151	142
340.3297	*	*	*	*	754059	168
341.1925	*	*	*	*	262931	248
341.8674	*	*	*	*	415421	356
344.8668	*	*	*	*	821418	160

350.8709	*	*	*	*	1240513	117
351.2233	*	*	*	*	216884	483
352.8624	*	*	*	*	562122	260
353.2013	*	*	*	*	390498	288
361.8630	*	*	*	*	302128	467
366.8466	*	*	*	*	228918	422
367.3573	*	*	*	*	298148	361
374.2441	*	*	*	*	287104	432
381.2342	*	*	*	*	203596	473
384.8589	*	*	*	*	412939	315
386.8524	*	*	*	*	179151	399
394.8495	*	*	*	*	365133	319
400.8672	*	*	*	*	2977211	39
409.3102	*	*	*	*	451418	224
410.8367	*	*	*	*	304478	339
416.8412	*	*	*	*	817711	172
417.2099	*	*	*	*	354630	261
421.2257	*	*	*	*	2301721	51
422.2288	*	*	*	*	570792	221
423.2245	*	*	*	*	275597	456
426.5552	*	*	*	*	807044	200
427.0561	*	*	*	*	250482	497
428.8500	*	*	*	*	289700	360
434.8543	*	*	*	*	531694	188
436.8368	*	*	*	*	304252	338
440.8600	*	*	*	*	1078031	109
444.8265	*	*	*	*	287791	368
446.5511	*	*	*	*	450658	283
450.8306	*	*	*	*	586296	173
456.8351	*	*	*	*	756102	153
460.3273	D-glucosyl-β1-1’-D-erythro-sphingosine	$C_{24}H_{47}NO_7$	[M-H]-	-1.3	4267940	28
461.3299	*	*	*	*	1184052	92
462.3323	*	*	*	*	237402	424
466.8083	*	*	*	*	203996	452
473.2814	*	*	*	*	1015332	105
473.3990	*	*	*	*	444919	230
474.2854	*	*	*	*	333586	303
479.2684	*	*	*	*	262524	370
479.5368	*	*	*	*	531206	235

482.3091	*	*	*	*	1173790	94
483.3112	*	*	*	*	356528	256
485.3375	18:1(d7) Lyso PE	C23H39D7NO7P	[M-H]-	-0.8	325094	322
486.3064	*	*	*	*	1384820	72
487.3095	*	*	*	*	412016	212
489.2758	Ubiquinone-4	C29H42O4	[M+Cl]-	-3.9	243232	413
493.2833	*	*	*	*	416153	237
496.3040	*	*	*	*	1543162	67
497.3072	*	*	*	*	453937	218
498.3016	*	*	*	*	593546	161
499.3037	*	*	*	*	198428	445
499.5332	*	*	*	*	379647	326
504.2914	*	*	*	*	342167	304
505.2861	*	*	*	*	355590	250
506.8316	*	*	*	*	719974	138
507.3025	*	*	*	*	341529	204
508.2922	*	*	*	*	291562	279
509.2861	*	*	*	*	217424	378
516.8017	*	*	*	*	227175	426
519.2985	*	*	*	*	489619	185
520.2970	*	*	*	*	199863	411
521.3168	*	*	*	*	321546	195
522.2916	*	*	*	*	243181	276
522.8048	*	*	*	*	255844	379
523.3033	*	*	*	*	138574	367
529.2830	*	*	*	*	90145	474
529.4615	*	*	*	*	1951070	53
530.4649	*	*	*	*	760643	135
531.4668	*	*	*	*	149549	453
532.5186	*	*	*	*	351806	296
533.3147	*	*	*	*	308208	292
534.8105	*	*	*	*	241856	316
535.3297	*	*	*	*	465052	164
535.5197	*	*	*	*	865292	118
536.3332	*	*	*	*	178587	469
536.5223	*	*	*	*	314031	309
540.8182	*	*	*	*	191350	428
543.3271	*	*	*	*	128084	477
546.8240	*	*	*	*	352207	259
547.3300	*	*	*	*	453339	190

549.3455	*	*	*	*	1302389	73
550.3509	PE 22:0	C27H54NO8P	[M-H]-	-0.9	522752	151
550.5190	C17 Ceramide	C35H68N03	[M-H]-	-2.5	16703896	6
551.3486	*	*	*	*	187355	390
551.5223	*	*	*	*	6454781	17
552.5240	*	*	*	*	1259850	66
553.5247	*	*	*	*	269536	332
554.2618	Leucine enkephalin	C28H37N5O7	[M-H]-	0.5	22327194	4
555.2647	Leucine enkephalin C13	C28H37N5O7	[M-H]-	-0.2	7952806	11
556.2696	*	*	*	*	1520423	54
556.7956	*	*	*	*	381953	258
557.2804	*	*	*	*	188004	242
561.3458	*	*	*	*	446544	209
561.4873	*	*	*	*	187161	444
562.8002	*	*	*	*	354544	277
563.3614	*	*	*	*	1338229	77
563.5508	*	*	*	*	894498	107
564.3628	*	*	*	*	373736	208
564.5538	*	*	*	*	373798	280
565.3560	*	*	*	*	166728	369
571.4960	*	*	*	*	243571	394
572.3741	*	*	*	*	149578	472
572.4998	*	*	*	*	256120	387
573.4941	*	*	*	*	210545	407
575.3616	*	*	*	*	443397	223
576.2437	*	*	*	*	4213755	15
576.3638	PE 24:0;OH	C29H58NO9P	[M-H20-H]-	-4.7	185072	459
577.2467	*	*	*	*	2101095	50
577.3768	*	*	*	*	726516	101
578.2493	*	*	*	*	416424	210
578.3799	PE 24:0	C29H58NO8P	[M-H]-	-4.8	380100	265
578.7762	*	*	*	*	207242	471
585.4996	*	*	*	*	217019	438
586.4958	*	*	*	*	6148365	19
587.4991	*	*	*	*	2413716	44
588.2739	*	*	*	*	199738	385
588.4952	*	*	*	*	2419154	43
589.4993	*	*	*	*	591239	111
590.5024	*	*	*	*	201399	436
591.3942	*	*	*	*	1152739	79

591.5834	*	*	*	*	280781	362
592.2172	*	*	*	*	158115	392
592.3978	PE 25:0	C30H60NO8P	[M-H]-	-1.0	264488	219
593.5191	*	*	*	*	222562	328
594.7755	*	*	*	*	223594	354
596.5251	*	*	*	*	742711	127
597.5249	*	*	*	*	497831	186
598.2254	*	*	*	*	3994870	26
598.5214	*	*	*	*	251529	307
599.2283	*	*	*	*	1371765	71
599.5196	*	*	*	*	165033	267
600.2310	*	*	*	*	302617	321
610.5416	*	*	*	*	255749	372
611.2815	*	*	*	*	397801	231
611.5367	*	*	*	*	486115	126
612.2209	*	*	*	*	380608	271
612.5300	*	*	*	*	161419	220
612.7950	*	*	*	*	455479	196
613.5154	*	*	*	*	1088494	61
614.2051	*	*	*	*	358820	225
614.5187	*	*	*	*	592567	147
615.2202	*	*	*	*	127216	381
615.5150	*	*	*	*	131628	440
620.2074	*	*	*	*	1772108	38
621.2107	*	*	*	*	832038	113
622.2191	*	*	*	*	195933	375
625.4059	Ubiquinone-6	C39H58O4	[M+Cl]-	4.8	181918	488
628.7682	*	*	*	*	177648	470
634.2030	*	*	*	*	231631	437
634.5015	*	*	*	*	271364	278
636.1851	*	*	*	*	313981	245
640.5512	*	*	*	*	651848	125
640.7737	*	*	*	*	166260	468
641.5545	*	*	*	*	328827	311
642.1895	*	*	*	*	1658828	60
643.1922	*	*	*	*	546697	175
649.4341	*	*	*	*	114051	374
656.4785	*	*	*	*	50709	493
658.1667	*	*	*	*	226035	382
660.4879	*	*	*	*	115648	371

662.2014	*	*	*	*	195105	458
662.4705	*	*	*	*	134664	419
662.7585	*	*	*	*	174673	486
664.1713	*	*	*	*	322055	286
664.4841	*	*	*	*	46854	433
666.5080	15:0-18:1(d7) PA	C36H61D7NaO8P	[M-H]-	-2.6	115843	481
674.4822	*	*	*	*	109160	343
676.4974	*	*	*	*	414447	189
677.4934	*	*	*	*	88462	310
681.6109	*	*	*	*	170399	495
686.5156	PE 33:0	C38H76NO8P	[M-H20-H]-	4.5	82659	434
688.4938	PE 32:1	C37H72NO8P	[M-H]-	2.2	506498	134
689.4954	CL 66:0	C75H146O17P2	[M-2H]2-	1.3	168860	268
690.5055	PE 32:0	C37H74NO8P	[M-H]-	-3.5	189116	291
693.4677	Ubiquinone-7	C44H66O4	[M+Cl]-	3.2	91525	450
696.1930	*	*	*	*	137219	494
702.5068	PE 33:1	C38H76NO9P	[M-H]-	-1.6	2085850	45
703.5101	CL 68:0	C77H150O17P2	[M-2H]2-	-0.1	518721	99
704.1896	*	*	*	*	278939	323
704.5164	*	*	*	*	288911	238
705.1906	*	*	*	*	96476	500
705.4829	*	*	*	*	79013	297
707.4767	*	*	*	*	83655	499
714.5236	*	*	*	*	101488	214
715.5674	*	*	*	*	99973	298
716.5367	*	*	*	*	420517	81
717.5390	*	*	*	*	152105	159
718.5166	*	*	*	*	183467	324
719.4861	PG 32:1	C38H73O10P	[M-H]-	-1.1	1002647	78
720.4872	*	*	*	*	478406	155
721.4946	*	*	*	*	324508	198
722.4835	*	*	*	*	103180	325
726.1719	*	*	*	*	1036029	100
727.1749	*	*	*	*	366607	251
728.5236	PE 35:1	C40H78NO8P	[M-H]-	< 0.1	349911	170
729.5269	CL 72:0	C81H154O17P2	[M-2H]2-	1.5	191501	384
730.5646	*	*	*	*	466445	70
731.5688	*	*	*	*	229077	140
732.5320	*	*	*	*	120724	336
733.5012	PG 33:1	C39H75O10P	[M-H]-	-1.8	2536994	37

734.5046	*	*	*	*	1384775	65
735.5082	*	*	*	*	421214	184
739.5362	*	*	*	*	156818	455
740.5447	15:0-18:1(d7) PG	C39H68D7O10P	[M-H]-	-2.3	1940051	48
741.5485	15:0-18:1(d7) PG C13		[M-H]-	-1.6	469160	114
742.1470	*	*	*	*	194894	404
742.5504	*	*	*	*	543204	121
743.5543	*	*	*	*	231374	263
744.5380	*	*	*	*	186129	320
744.5967	*	*	*	*	143794	408
745.5063	PG 34:2	C40H75O10P	[M-H]-	5.1	266968	229
746.5137	*	*	*	*	181315	211
747.5164	PG 34:1	C40H77O10P	[M-H]-	-2.4	1284500	56
748.1541	*	*	*	*	468259	157
748.5199	*	*	*	*	630569	110
748.6817	*	*	*	*	163595	464
749.1583	*	*	*	*	196609	359
749.5230	*	*	*	*	248775	346
750.6969	*	*	*	*	423358	141
751.6990	*	*	*	*	364874	254
752.7033	*	*	*	*	141481	461
753.5445	15:0-18:1(d7) PS	C39H67D7NO10P	[M-H]-	3.7	147319	448
755.4771	*	*	*	*	63720	463
756.5556	PE 37:2	C42H80NO8P	[M-H]-	0.9	360832	174
757.5587	*	*	*	*	103943	352
758.6132	*	*	*	*	943932	91
759.5172	PG 35:2	C41H77O10P	[M-H]-	-1.3	522788	143
759.6169	*	*	*	*	258147	206
760.5151	PS 34:1	C40H76NO10P	[M-H]-	2.2	715252	112
761.5300	Ubiquinone-8	C49H74O4	[M+Cl]-	2.5	1056142	55
762.5341	*	*	*	*	528937	116
763.5372	*	*	*	*	258362	345
764.7047	*	*	*	*	139308	240
765.7061	*	*	*	*	100067	377
766.6923	*	*	*	*	1031008	96
767.6957	*	*	*	*	546465	171
768.7026	*	*	*	*	199471	330
770.1365	*	*	*	*	100433	449
772.5382	*	*	*	*	85903	201
773.5331	PG 36:2	C42H79O10P	[M-H]-	-0.9	979601	75

774.5435	*	*	*	*	340910	98
775.5477	PG 36:1	C42H81O10P	[M-H]-	-2.3	439069	154
776.5502	*	*	*	*	92618	396
776.7125	*	*	*	*	583267	149
777.7159	*	*	*	*	330959	269
778.7259	*	*	*	*	667971	133
779.7294	*	*	*	*	249533	243
780.7142	*	*	*	*	292266	249
786.5234	*	*	*	*	191051	181
787.5491	PG 37:2	C43H81O10P	[M-H]-	-0.5	864861	90
788.5679	*	*	*	*	619246	74
789.5737	*	*	*	*	314172	148
790.5736	*	*	*	*	63515	415
792.7332	*	*	*	*	264151	199
793.7335	*	*	*	*	74169	314
794.7230	*	*	*	*	327963	233
795.7254	*	*	*	*	172839	462
796.5071	*	*	*	*	33679	479
797.6194	*	*	*	*	105956	443
798.6246	*	*	*	*	102432	363
800.5783	PS 38:0	C44H86NO10P	[M-H20-H]-	-2.7	124003	167
801.5716	*	*	*	*	203278	246
802.5864	*	*	*	*	186071	282
804.7429	*	*	*	*	622293	139
805.7529	*	*	*	*	217888	162
806.7592	*	*	*	*	1259913	46
807.7626	*	*	*	*	621393	80
808.7639	*	*	*	*	340648	213
810.1501	*	*	*	*	123778	348
812.6109	*	*	*	*	75836	353
813.5995	*	*	*	*	401836	179
814.6003	*	*	*	*	244201	253
816.1548	*	*	*	*	149764	492
816.6198	*	*	*	*	545835	156
816.6731	*	*	*	*	175510	388
817.6230	*	*	*	*	166762	335
818.7599	*	*	*	*	135657	347
819.7742	*	*	*	*	95159	318
820.7747	*	*	*	*	2539307	22
821.7781	*	*	*	*	1690398	36

822.7809	*	*	*	*	685645	119
823.7831	*	*	*	*	114072	482
824.7220	*	*	*	*	114859	287
828.5609	15:0-18:1(d7) PI	C42H72D7O13P	[M-H]-	-1.9	298826	236
832.1361	*	*	*	*	241751	308
832.7746	*	*	*	*	732785	129
833.7888	*	*	*	*	277605	132
834.7913	*	*	*	*	1271736	69
835.7947	*	*	*	*	460867	128
836.7962	*	*	*	*	116955	365
844.6960	*	*	*	*	70077	380
848.8062	*	*	*	*	5153078	14
849.8094	*	*	*	*	1992652	29
850.8121	*	*	*	*	779569	82
851.8153	*	*	*	*	259580	357
861.8364	*	*	*	*	206209	398
862.6956	*	*	*	*	374815	234
862.8331	*	*	*	*	84468	418
863.6981	*	*	*	*	235338	313
864.7039	*	*	*	*	106709	466
876.7106	*	*	*	*	384041	222
877.7132	*	*	*	*	136205	391
890.7266	*	*	*	*	141549	337
921.6613	*	*	*	*	558827	145
922.6641	*	*	*	*	170584	274
943.6431	*	*	*	*	174659	373
946.6647	*	*	*	*	120909	480
979.6627	*	*	*	*	93972	366
980.6663	*	*	*	*	80374	421
1011.8536	*	*	*	*	292156	299
1012.8571	*	*	*	*	178290	417
1013.1001	*	*	*	*	89536	465
1015.6039	*	*	*	*	73806	405
1040.6884	4-Amino-4-deoxy-alpha-L-arabinopyranosyl di-trans,octa-cis-undecaprenyl phosphate	C61H100NO8P	[M+Cl]-	0.3	13733	397
1050.8751	*	*	*	*	84427	485
1102.0471	*	*	*	*	1125971	64
1103.0503	*	*	*	*	1039250	83
1104.0533	*	*	*	*	370978	192
1109.5321	*	*	*	*	266605	205

1110.5350	*	*	*	*	259991	272
1131.5137	*	*	*	*	224194	177
1132.5168	*	*	*	*	253121	285
1133.5209	*	*	*	*	107671	490
1138.0242	*	*	*	*	504789	166
1139.0280	*	*	*	*	272612	217
1140.0261	*	*	*	*	264302	257
1141.0282	*	*	*	*	124860	414
1153.4965	*	*	*	*	145940	284
1154.4993	*	*	*	*	168530	412
1163.0652	*	*	*	*	111562	400
1163.8417	*	*	*	*	186759	226
1164.0646	*	*	*	*	91286	435
1164.8455	*	*	*	*	125538	301
1175.4783	*	*	*	*	92739	403
1176.4816	*	*	*	*	67204	454
1177.8608	*	*	*	*	66845	420
1191.8871	*	*	*	*	59507	442
1194.8368	*	*	*	*	95275	312
1195.8396	*	*	*	*	122460	489

* feature characteristics marked with asterisks were not able to be unambiguously identified based on thresholding parameters

Table S4: Significant feature table for TY04 and NHL17 comparison

m/z	Tentative ID	Fold Change	p-Value	log2(Fold Change)	-log10(p-Value)
143.1073	Octanoic Acid	2.7134	0.00023295	1.4401	3.6327
197.1541	*	3.085	0.0069244	1.6252	2.1596
199.1334	Dodecanoic Acid	2.4658	0.0013461	1.3021	2.8709
211.1693	*	4.1476	0.0015216	2.0523	2.8177
213.149	*	2.0999	0.0057066	1.0703	2.2436
213.1851	*	3.9014	0.00051082	1.964	3.2917
214.1893	*	3.9544	0.00029775	1.9835	3.5261
215.1649	*	2.0007	0.037809	1.0005	1.4224
225.1855	Tetradecenoic Acid	6.262	0.00036116	2.6466	3.4423
226.189	Tetradecenoic Acid C13	5.588	0.00061323	2.4823	3.2124
227.2009	Tetradecanoic Acid	2.5293	0.0035713	1.3388	2.4472
228.2048	Tetradecanoic Acid C13	2.507	0.00042881	1.326	3.3677
229.1805	*	2.4273	0.0028301	1.2793	2.5482
239.2011	*	2.6694	0.0010465	1.4165	2.9803
241.1802	*	2.2345	0.0051121	1.1599	2.2914
254.2202	*	3.0261	0.0012471	1.5974	2.9041
479.2684	*	2.3861	0.024234	1.2547	1.6156
493.2833	*	2.425	0.030207	1.278	1.5199
505.2861	*	2.0521	0.0033878	1.0371	2.4701
507.3025	*	2.5351	0.0011266	1.342	2.9482
519.2985	*	2.8167	0.0007549	1.494	3.1221
533.3147	*	2.7177	0.0017287	1.4424	2.7623
536.3332	*	0.48554	0.0011291	-1.0423	2.9473
572.3741	*	2.058	0.010742	1.0413	1.9689
662.4705	*	0.46019	0.00039178	-1.1197	3.407
686.5156	PE 33:0	3.5691	0.00027277	1.8356	3.5642
690.5055	PE 32:0	0.40653	0.032091	-1.2986	1.4936
693.4677	Ubiquinone-7	0.19413	6.20E-05	-2.3649	4.2073

703.5101	CL 68:0	0.45852	0.0012388	-1.1249	2.907
707.4767	*	0.34626	0.003905	-1.5301	2.4084
719.4861	PG 32:1	0.11242	0.0037629	-3.153	2.4245
720.4872	*	0.14343	0.00056257	-2.8016	3.2498
721.4946	*	0.13373	0.00020737	-2.9026	3.6832
722.4835	*	0.30906	2.96E-05	-1.694	4.5283
733.5012	PG 33:1	0.29494	5.65E-05	-1.7615	4.2478
734.5046	*	0.31208	5.11E-05	-1.68	4.292
735.5082	*	0.34376	0.0040416	-1.5405	2.3935
748.6817	*	3.0114	0.015709	1.5904	1.8038
759.5172	PG 35:2	2.4377	0.0002289	1.2855	3.6403
764.7047	*	3.6988	0.0031332	1.8871	2.504
765.7061	*	3.6377	0.046284	1.863	1.3346
772.5382	*	2.6728	0.028572	1.4184	1.5441
773.5331	PG 36:2	2.5307	0.018642	1.3395	1.7295
776.7125	*	5.8427	0.00048141	2.5466	3.3175
777.7159	*	4.4134	0.0010443	2.1419	2.9812
778.7259	*	2.4582	0.00031706	1.2976	3.4989
779.7294	*	2.7441	0.038108	1.4563	1.419
780.7142	*	2.0208	0.0071987	1.0149	2.1427
787.5491	PG 37:2	5.1053	0.00023961	2.352	3.6205
801.5716	*	3.8232	0.00022293	1.9348	3.6518
804.7429	*	3.0862	0.0017257	1.6258	2.763
805.7529	*	2.6995	0.0031378	1.4327	2.5034
1194.8368	*	0.37915	0.00031817	-1.3992	3.4973
1195.8396	*	0.39889	0.015978	-1.326	1.7965

* indicates feature not able to be unambiguously identified based on thresholding parameters

Table S5: Significant feature table for TY04 and TY05 comparison

m/z	Tentative ID	Fold Change	p-Value	log2(Fold Change)	-log10(p-Value)
59.0139	Acetic acid	14.22	5.25E-05	3.8298	4.2796
159.065	*	3.8093	0.0012883	1.9295	2.89
183.0095	*	0.42891	0.00049531	-1.2213	3.3051
197.1541	*	15.286	7.31E-05	3.9341	4.136
198.1564	*	6.0225	7.51E-05	2.5904	4.1241
199.1695	Dodecanoic acid (C12:0)	11.165	0.0079865	3.4809	2.0976
200.1731	Dodecanoic acid (C12:0) C13	11.069	0.0083455	3.4684	2.0785
201.1759	*	8.6801	0.015004	3.1177	1.8238
213.149	*	35.252	0.00029402	5.1396	3.5316
215.1649	*	184.76	0.0010515	7.5295	2.9782
216.1684	*	128.27	0.00089285	7.003	3.0492
225.1855	Tetradecenoic Acid (C14:1)	50.85	0.0001126	5.6682	3.9485
226.189	Tetradecenoic Acid (C14:1) C13	45.458	9.87E-05	5.5065	4.0055
227.2009	Tetradecanoic Acid (C14:0)	4.3104	0.0070938	2.1078	2.1491
228.2048	Tetradecanoic Acid (C14:0) C13	4.2428	0.010173	2.085	1.9925
241.1802	*	33.127	0.00057415	5.0499	3.241
242.1793	*	2.467	0.0082434	1.3028	2.0839
243.196	Hydroxymyristic acid (C14:0;OH)	3.4734	4.38E-05	1.7963	4.3585
244.1993	Hydroxymyristic acid (C14:0;OH) C13	3.257	1.68E-05	1.7035	4.7758
255.2323	Hexadecanoic acid (C16:0)	0.46728	0.00071565	-1.0977	3.1453
256.2357	Hexadecanoic acid (C16:0) C13	0.46327	0.00068294	-1.1101	3.1656
257.2385	*	0.47218	0.0001725	-1.0826	3.7632

265.1472	*	0.34699	0.0055181	-1.527	2.2582
266.1503	*	0.32096	0.015242	-1.6395	1.8169
267.1481	*	0.49697	0.0052493	-1.0088	2.2799
267.2324	Avocadene	0.45879	0.00809	-1.1241	2.092
268.236	*	0.4811	0.0094077	-1.0556	2.0265
291.1601	*	0.47087	0.017846	-1.0866	1.7485
293.1783	*	0.44633	0.02947	-1.1638	1.5306
294.1815	*	0.47301	0.030047	-1.08	1.5222
297.1526	*	0.33057	0.0015074	-1.597	2.8218
298.1562	*	0.32968	0.0034957	-1.6009	2.4565
311.1682	*	0.34263	0.0020331	-1.5453	2.6919
312.1715	*	0.3328	0.0021285	-1.5873	2.6719
313.1671	*	0.41134	0.0019608	-1.2816	2.7076
318.2063	*	0.49578	0.002157	-1.0122	2.6661
325.1837	*	0.33142	0.0044263	-1.5933	2.354
326.1873	*	0.34799	0.0058607	-1.5229	2.232
339.1992	*	0.33545	0.0047581	-1.5758	2.3226
340.2026	*	0.37042	0.012842	-1.4328	1.8914
505.2861	*	6.9249	0.00053483	2.7918	3.2718
507.3025	*	5.4009	0.002965	2.4332	2.528
509.2861	*	5.461	0.047868	2.4492	1.32
535.3297	*	0.34482	0.0043191	-1.5361	2.3646
536.3332	*	0.358	3.89E-05	-1.482	4.4106
549.3455	*	0.35222	0.00016918	-1.5055	3.7717
550.3509	PE 22:0	0.48805	0.0017339	-1.0349	2.761
551.3486	*	0.46702	5.24E-05	-1.0984	4.2805
563.3614	*	0.48699	0.036151	-1.038	1.4419
572.3741	*	0.42578	0.026107	-1.2318	1.5832
577.3768	*	0.40045	0.0023539	-1.3203	2.6282
578.3799	*	0.45045	0.0018134	-1.1506	2.7415
660.4879	*	3.0809	0.006444	1.6233	2.1908
662.4705	*	0.49118	0.00061735	-1.0257	3.2095
676.4974	*	14.086	0.00098614	3.8161	3.0061
677.4934	*	7.4554	0.00025182	2.8983	3.5989
686.5156	PE 33:0	22.206	0.0001843	4.4729	3.7345
688.4938	PE 32:1	0.39103	0.0031886	-1.3547	2.4964
689.4954	CL 66:0	0.39814	0.044537	-1.3287	1.3513
693.4677	Ubiquinone-7	0.14589	4.66E-05	-2.7771	4.3313
702.5068	PE 33:1	0.3389	0.0012367	-1.5611	2.9077
703.5101	CL 68:0	0.31983	0.00042077	-1.6446	3.376

705.4829	*	0.31401	0.02257	-1.6711	1.6465
707.4767	*	0.32332	0.0044082	-1.629	2.3557
718.5166	*	2.9349	0.0022952	1.5533	2.6392
719.4861	PG 32:1	0.073868	0.0031267	-3.7589	2.5049
720.4872	*	0.093518	0.00045112	-3.4186	3.3457
721.4946	*	0.10092	0.00018978	-3.3088	3.7218
722.4835	*	0.34911	3.30E-05	-1.5182	4.4821
732.532	*	2.8805	0.045444	1.5263	1.3425
733.5012	PG 33:1	0.12541	2.58E-05	-2.9953	4.5884
734.5046	*	0.45204	0.00015429	-1.1455	3.8117
744.538	*	3.6506	0.00047789	1.8681	3.3207
748.6817	*	8.7266	0.0040655	3.1254	2.3909
750.6969	*	7.1218	0.017901	2.8322	1.7471
751.699	*	7.3934	0.0058237	2.8862	2.2348
752.7033	*	5.9766	0.0071954	2.5793	2.1429
755.4771	*	0.48362	0.0061671	-1.048	2.2099
756.5556	PE 37:2	2.3584	0.014746	1.2378	1.8313
757.5587	*	2.1455	0.0099003	1.1013	2.0044
766.6923	*	92.514	0.00084361	6.5316	3.0739
767.6957	*	83.956	0.00052374	6.3916	3.2809
768.7026	*	20.245	0.0012921	4.3395	2.8887
773.5331	PG 36:2	4.2983	0.001151	2.1038	2.9389
774.5435	*	2.2707	0.0065631	1.1832	2.1829
775.5477	PG 36:1	2.0448	0.048064	1.032	1.3182
776.7125	*	24.39	0.0015821	4.6082	2.8008
777.7159	*	19.16	0.0021087	4.26	2.676
778.7259	*	2.8811	0.0027732	1.5266	2.557
779.7294	*	3.3363	0.013979	1.7382	1.8545
787.5491	PG 37:2	4.9214	0.015103	2.2991	1.8209
794.723	*	2.737	0.00030824	1.4526	3.5111
795.7254	*	2.5152	0.00062495	1.3307	3.2042
801.5716	*	2.5647	0.01378	1.3588	1.8607
807.7626	*	0.29865	0.0025018	-1.7435	2.6018
808.7639	*	0.35442	0.0017422	-1.4965	2.7589
816.6731	*	0.40665	0.01613	-1.2981	1.7924
818.7599	*	0.30303	0.001137	-1.7225	2.9442
819.7742	*	0.39112	0.011351	-1.3543	1.945
820.7747	*	0.47828	0.0060595	-1.0641	2.2176
822.7809	*	0.4631	0.017024	-1.1106	1.7689
844.696	*	0.41242	0.02368	-1.2778	1.6256

848.8062	*	0.49723	0.016499	-1.008	1.7825
862.6956	*	0.30898	0.022097	-1.6944	1.6557
863.6981	*	0.30508	0.0050624	-1.7127	2.2956
864.7039	*	0.45829	0.043442	-1.1257	1.3621
876.7106	*	0.27057	0.0063511	-1.8859	2.1972
877.7132	*	0.26746	0.00043258	-1.9026	3.3639
890.7266	*	0.32571	0.0026522	-1.6184	2.5764
1163.8417	*	0.32567	0.00031178	-1.6185	3.5062
1164.8455	*	0.35188	0.0017923	-1.5068	2.7466
1194.8368	*	0.2002	0.00013835	-2.3205	3.859
1195.8396	*	0.35386	0.011199	-1.4987	1.9508

* indicates feature not able to be unambiguously identified based on thresholding parameters
